# The regulation of AsfR on tmRNA expression mediates bacterial motility and virulence in *Aeromonas veronii*

**DOI:** 10.1080/21505594.2025.2602247

**Published:** 2025-12-10

**Authors:** Huimin Chang, Yuan Tong, Shijie Gao, Xiaoli Jiang, Xiang Ma, Hong Li, Yanqiong Tang, Juanjuan Li, Hongqian Tang, Min Lin, Zhu Liu

**Affiliations:** a Faculty of Animal Science and Technology, Yunnan Agricultural University, Kunming, China; b School of Life and Health Sciences, Hainan University, Haikou, China; c Biotechnology Research Institute, Chinese Academy of Agricultural Sciences, Beijing, China

**Keywords:** AsfR, tmRNA, motility, antioxidant capacity, virulence

## Abstract

Transfer messenger RNA (tmRNA), a key component of the trans-translation system, plays an essential role on the virulence of pathogenic bacteria. However, the upstream regulatory mechanisms that regulate tmRNA expression remain largely unexplored. In this study, AraC superfamily regulator (AsfR) was found to directly interact with the promoter of *ssrA* gene, which encodes tmRNA. Co-transformation of the reporter construct, consisting of tmRNA promoter fused to enhanced green fluorescent protein (eGFP), alongside an AsfR expression vector, resulted in increased fluorescence, indicating that AsfR positively regulates mRNA expression. Consistently, the transcription level of tmRNA was significantly decreased in Δ*asfR* compared with WT of *A. veronii* by quantitative real-time PCR (RT-qPCR) analyses. The Δ*asfR* and Δ*tmRNA* mutants exhibited significantly reduced motility and biofilm formation. Reduced transcription of the flagellar gene *fliE* in both mutants suggests that the AsfR/tmRNA axis may regulate these processes via *fliE*. Furthermore, deletion of *asfR* and tmRNA impairs oxidant resistance and pathogenicity, resulting in growth inhibition in *A. veronii*. This study elucidates the regulatory role of the AsfR-tmRNA pathway in flagellar motility, biofilm formation, and antioxidant capacity, all of which contribute to bacterial virulence and provide potential targets for the treatment of bacterial infections.

## Introduction

In recent years, the genus *Aeromonas* has been isolated from various environments, including food, aquatic products, and the human body. Among these species, *Aeromonas veronii* is regarded as a significant pathogen [1–5] responsible for a wide range of human diseases, including septicemia [6], diarrhea [7], fasciitis [8], and meningitis [9]. These diseases pose a public health challenge as the prevalence of *A. veronii* infections continues to increase globally.

The trans-translation system is the primary ribosome rescue mechanism in bacteria [10,11], with transfer-messenger RNA (tmRNA, encoded by *ssrA* gene) serving as its essential component. The malfunction of trans-translation results in a variety of phenotypic defects, including motility, amino acid metabolism and virulence [11–15]. For instance, the ∆*tmRNA* mutants exhibit reduced motility in several bacterial species, including *Bacillus subtilis* [15,16], *Escherichia coli* [17] and *Caulobacter crescentus* [18]. In *Bacillus subtilis*, the absence of tmRNA impairs amino acid anabolic processes [15]. High-throughput screening first identified acylaminooxadiazole KKL-series compounds as inhibitors of trans-translation [19]. MBX-4132 of KKL series efficiently cleared multidrug-resistant *Neisseria gonorrhoeae* in a mouse infection model [20]. KKL-1005 as a small-molecule inhibitor that targets ribosomal protein bL12, thereby specifically blocking tmRNA – SmpB – mediated trans-translation and exhibiting anti-tubercular activity [21]. Bacterial virulence is often closely associated with flagellar function, as flagella play a critical role in enabling pathogenic bacteria to reach optimal niches and invade and colonize host tissues [22]. Furthermore, flagella-mediated motility contributes to both initial surface attachment and subsequent biofilm formation [23]. These studies suggest that trans-translation, mediated by tmRNA, represents a promising target for combating multidrug resistance and disease treatment.

The current research primarily addresses the downstream regulatory functions of trans-translation. However, upstream regulation, particularly the role of transcriptional factors in controlling tmRNA expression, remains largely unexplored. To address this gap, transcriptional factor prediction tools (MEME and Prodoric) and RNA-Seq analyses were employed to search for potential upstream regulators of tmRNA. The AsfR (AraC superfamily regulator) has been identified as a potential regulatory factor. The AraC family, one of the largest groups of regulatory proteins, controls various processes including virulence, stress responses and amino acid metabolism [24,25]. For example, the AraC family regulator RamA influences virulence and amino acid biosynthesis by repressingtranscription of downstream genes such as *invF*, *sipC*, *sopB* and *ssrA* [25]. This study aimed to investigate the regulatory role of AsfR as a novel transcriptional regulator of tmRNA and its subsequent effects on flagellar motility, antioxidant capacity and virulence. Our findings shed light on the underlying mechanisms of bacterial pathogenesis and have implications for the development of effective strategies to combat infectious diseases in humans and animals.

## Materials and methods

### Strains and plasmids

The strains, plasmids and primers used in this study were listed in Supplementary Tables S1, S2 and S3, respectively. It is noteworthy that *Aeromonas veronii* C4 was isolated from the diseased fish [26]. Bacteria were cultured in LB/M9 broth or on LB agar plates at 30°C.The final concentration of 0.1 mM isopropyl-1-thio-β-D-galactopyranoside (IPTG) was added to induce the T7 promoter expression. Each strain was supplemented with the corresponding antibiotics, including 50 µg/mL ampicillin (Amp),50 µg/mL kanamycin (Kan), 12.5 µg/mL tetracycline (Tet), 25 µg/mL chloramphenicol (Chl) and 12.5 µg/ml streptomycin (Str).

### The prediction of upstream transcription factors of tmRNA gene

The potential conserved motif binding to the tmRNA promoter was predicted using two distinct methods. Initially, multiple sequences of tmRNA promoters were input into the Motif Discovery tool of the MEME Web platform to infer the motif [27]. Subsequently, the inferred motif was compared with known motifs cataloged in the prokaryotic database CollecTF by employing the motif annotation tool Tomtom to define similarities [28]. Alternatively, the promoter regions were submitted to the PRODORIC database for Virtual Footprint analysis to infer the putative motif. Ultimately, the resultant conserved motifs were aligned with the protein database of *A. veronii* C4 utilizing the NCBI BLAST tool. The transcription factors associated with the tmRNA promoter were identified by analyzing the ranked significance of matches between the input protein sequence and those of *A. veronii* C4.

### Gene knock-out

Mutants of *A. veronii* C4 were constructed using the homologous recombination method [26,29]. Briefly, the upstream and downstream homologous arms of the target genes were cloned into the pRE112 plasmid. Subsequently, the recombinant plasmid was electrically transformed into *E. coli* WM3064 for selection using diaminopimelic acid (DAP), followed by transfer into *A. veronii* via bacterial conjugation. The knockout strain was screened on Luria-Bertani (LB) agar plates containing 8% sucrose. To construct the complemented strain, the target gene was integrated into the pBBR plasmid and transformed into *A. veronii* knockout. The *tmRNA* knockout and the *asfR* knockout was constructed previously [30,31], while both the *asfR*- *tmRNA* deletion and complemented strains were generated in the present study.

### Bacterial one-hybrid

The *E. coli* XL1-Blue MRF’ strain was used topropagate pTRG, pBX-cmT or their derivatives. Co-transformation including both pTRG and pBX-cmT derivatives were selected in M9^+^ His-Dropout medium containing 3-amino-1,2,4-triazole (3-AT), streptomycin, tetracycline, and chloramphenicol [32]. After a series of 10-fold gradient dilutions, the transformants were dropped on the culture plates containing different concentration of 3AT (0 mM, 2.5 mM, 5 mM, 10 and 12 mM) and then incubated at 37°C for 24 h.

### Enhanced GFP fluorescent assays

The promoter region of tmRNA was fused with enhanced green fluorescent protein (eGFP) to serve as a reporter vector. This construct was co-transformed with the *asfR* expression plasmid into *E. coli* XL1-Blue MRF’ Reporter. The resulting transformant was cultured in medium supplemented with the corresponding antibiotics at 30°C overnight and subsequently reinoculated into fresh medium until a concentration of 2 × 10^6^ CFU/mL. The samples were placed in a 96-well plate and analyzed using a Synergy H1 Multi-Mode Reader (BioTek, USA). Fluorescence was detected at 485 nm excitation and 525 nm emission. All measurements were performed in triplicate.

### RT-qPCR assay

RNA extraction was performed after culture in the stationary phase. The reverse transcription reaction was performed according to the protocol of the HiScript®IIQ RT Super Mix Kit (Vazyme, China), of which 500 ng RNA was used as the template and gDNA wiper Mix was used to remove genomic DNA. The quantitative real-time PCR (RT-qPCR) assay was conducted according to the instructions provided with the ChamQ SYBR Color qPCR Master Mix Kit (Vazyme, China). Cycle threshold (CT) values were determined using a Roche LightCycler® 96 instrument (Roche Diagnostics, Switzerland). Relative gene expression was calculated using the quantification cycle (∆∆Cq) method [33,34].

### Transcriptome sequencing and analysis

Transcriptome analysis was performed by BGI Genomics Co., Ltd. Total RNA was extracted using TRIzol reagent, and rRNA was removed using the RiboX rRNA removal kit. Following RNA extraction, RNA library was constructed using the MGIEasy RNA library prep kit V2, and sequencing was performed on an BGISEQ-500 sequencer in SE50 mode [35]. Clean reads were aligned to reference genes using Bowtie2 [36], and gene expression levels were quantified with RSEM software package [37]. For gene expression analysis, sample correlation was evaluated using the cor function in R software, with Pearson’s correlation employed to assess the linear relationship between data from the two groups. Principal component analysis (PCA) was performed using the ade4 software package in R. Hierarchical clustering was implemented using the hclust function in the R software, with Euclidean distance as the distance metric. The data were deposited in the NCBI database under accession number GSE120603 (https://www.ncbi.nlm.nih.gov/geo/query/acc.cgi?acc=GSE120603). These data were the same as those in our previous publication [38], but were subjected to further in-depth analysis.

### Metabolome sequencing and analysis

Metabolomic assays were conducted by Shanghai BioTree Biotech. The metabolites were redissolved by adding 100 μL of extraction solution (acetonitrile and water, 1:1, v/v), followed by high-speed centrifugation. The samples were then filtered and subjected to UPLC-MS/MS analysis. PCA was employed to identify the overall metabolic differences between the sample groups using the SIMCA14.1 software package (V14.1, Sartorius Stedim Data Analytics AB, Umea, Sweden). Differential metabolites were evaluated using orthogonal projections to latent structure discriminantsanalysis (OPLS-DA). To refine this analysis, the variable importance in projection (VIP) values for the first principal component were calculated, and metabolites with VIP values greater than 1 were selected. These variables were subsequently evaluated using the Student’s t-test, with a significance threshold of *p* > 0.05, and those not meeting this criterion were excluded from the comparison. In addition, KEGG and MetaboAnalyst were used to search for metabolite pathways. The data were available in the Metabolights repository (accession code: MTBLS1191; https://www.ebi.ac.uk/metabolights/). These datasets were identical to those used in our original publication [38], but have undergone additional advanced computational analysis.

### Motility test

Motility tests were performeded on swimming and swarming plates containing 0.3% and 0.5% agarose, respectively [39]. After the bacterial solution was diluted to 1 × 10^7^ CFU/mL, 2 uL of the sample was spotted onto the plates and incubated at 30 °C for 6 h.

### Biofilm assay

The strain was cultured in medium supplemented with the appropriate antibiotics at 30°C overnight and subsequently inoculated in fresh medium until reaching a final concentration of 2 × 10^6^ CFU/mL. Samples of 200 μL were cultured in a 96-well plate for 48 h. Biofilm-forming abilities were evaluated using crystal violet staining [40], followed by absorbance measurement at 570 nm using a Synergy H1 Multi-Mode Reader (BioTek, USA).

### Bacterial growth inhibition of oxidative stress

For H_2_O_2_ treatment, the stock solutions were diluted in PBS to obtain final H_2_O_2_ concentrations of 50, 125, 250, 500 μM. After treatment with H_2_O_2_ for 30 min at 30°C, 10 μL of each treated bacterial samples (OD600nm = 1) were transferred to 90 µL of sterilized PBS, resulting in the first 10-fold dilution. Two microliters of the bacterial suspension were directly spotted on an M9 agar plate. Consecutive 10-fold dilutions were performed from which 2 µl was spotted each time on LB agar plates. The agar plates were placed in an incubator at 37 °C overnight and the resulting plates were imaged with the ChemiDoc XRS+ Imagers (BioRad, USA).

### AsfR protein purification

*E. coli* BL21 expressing pET-28a-AsfR was cultured overnight and then inoculated into fresh medium until it reached OD_600_ of 0.4–0.6. Following this, the final concentration of 0.1 mM IPTG was added to induce protein expression at 30 °C for 3 h. The cells were lysed by ultrasonication and loaded onto a Nickel-Immobilized Metal Affinity Chromatography (NI-IDA) column. The AsfR protein was eluted with 250 mM imidazole solution (50 mM HEPES,300 mM NaCl,pH7.5), followed by overnight dialysis in HEPES solution (50 mM HEPES,300 mM NaCl,250 mM imidazole, pH7.5). Protein purity was determined by sodium dodecyl sulfate-polyacrylamide gel electrophoresis (SDS-PAGE) with Coomassie bright blue staining, and imaging was visualized using ChemiDoc XRS^+^ Imagers (BioRad, USA). The protein concentration was determined using a BCA protein assay kit (Beyotime Biotechnology, China).

### Electrophoretic mobility-shift assays (EMSA)

EMSA was performed according to a previously described protocol with minor revision [41]. Briefly, the promoter fragment of *ssrA* gene encoding the tmRNA was labeled with 6-FAM, and 40 ng of the promoter was mixed with varying concentrations of AsfR protein. The reaction mixture was incubated at 30°C for 1 h in a solution consisting of 20 mM HEPES, 100 mM NaCl, 1 mM DTT, 5 mM MgCl_2_, at pH 7.5. After the samples was run in 6% native polyacrylamide gel, fluorescence imaging was performed using a Typhoon FLA 9500 (GeneralElectric, USA).

### Molecular docking and interaction assay

The 3D structure of the the AsfR protein and tmRNA promoter was predicted using AlphaFold3 [42]. Protein – DNA docking was performed using HADDOCK2.4 in protein – DNA mode, guided by predicted DNA-binding residues and promoter sequences. Docking results were clustered by RMSD and ranked by HADDOCK score [43]. The top-ranked models were visualized in PyMOL, where protein – DNA interfaces were examined. Hydrogen bonds and intermolecular distances were measured using PyMOL’s distance and find_pairs functions to identify key contacts stabilizing the complex.

### Transmission electron microscope (TEM)

The suspensions of *A. veronii* derivatives, including Δ*asfR*, Δ*tmRNA* and Δ*asfR*Δ*tmRNA*, were dropped onto a copper grid with a carbon film for 5 min, followed by elimination of the excess liquid with filter paper. Subsequently, 2% phosphotungstic acid was added and left for 1–2 minutes. The grid was washed twice with ddH_2_O and dried at room temperature. The images were observed using an HT7800 transmission electron microscope (Hitachi, Japan). Flagella structure were directly visualized by TEM imaging [44], and quantified manually from randomly selected fields. For each strain, at least 30 individual bacteria were analyzed to determine the distribution of flagella.

### Animal studies and ethical statement

Male Kunming mice (8 weeks old, weighing 27 ± 1 g) were obtained from Slykejingda Laboratory Animal Co. LTD (Changsha City, Hunan Province, China). The mice were housed under a 12/12-hour light/dark cycle with ad libitum access to water and standard commercial feed (Haikou city, Hainan Province, China) in a pathogen-free condition with a controlled temperature (23 ± 3°C). Mice were euthanized by cervical dislocation performed by a trained operator in accordance with veterinary guidelines approved by the American Veterinary Medical Association [45], ensuring rapid loss of consciousness and absence of reflexes immediately after the procedure. All animal experiments were conducted in accordance with the instructions of the Animal Management and Ethics Committee of Hainan University (HNUAUCC-2023–00141). All animal infection protocols strictly adhered to the National Guiding Principles for the Welfare of Laboratory Animals. This study strictly adhered to the ARRIVE guidelines (Animal Research: Reporting of *In Vivo* Experiments) to ensure comprehensive reporting of animal research. The completed ARRIVE checklist was provided in Supplementary Materials.

### Virulence assay in mice model

Overnight cultures of *A. veronii* derivatives including Δ*asfR*, Δ*tmRNA*, Δ*asfR*Δ*tmRNA* and the corresponding complementation strains were diluted in phosphate-buffered saline PBS (10 mM, pH 7.4) to a concentration of 6 × 10^7^ CFU/mL. Subsequently, 100 μL of the dilution was intraperitoneally injected into 5-week-old female KM mice. After the mice were euthanized 8 h post-infection, the Organs were harvested, washed three times with PBS to remove surface bacteria, homogenized in PBS, serially diluted, and plated on LB agar for bacterial enumeration after overnight incubation [46]. Data were obtained from five independent biological replicates.

### Infection assay in L929 fibroblasts

Mouse L929 fibroblasts were cultured in RPMI 1640 medium supplemented with 10% fetal bovine serum (FBS) and appropriate antibiotics (100 units/ml of penicillinG and 100 μg/ml of streptomycin) in a 5% CO_2_ atmosphere at 37°C. Cells were plated at a density of 1 × 10^5^ cells per well in a 96-well dish and infected at a MOI of 1:10 with *A. veronii* derivative at 37°C with 5% CO_2_ for 2 h. Extracellular bacteria were eliminated by treatment with 50 μg/mL gentamicin for 1 h, followed by three washes with 10 mM PBS (pH 7.4). Subsequently, the cells were overlaid with 0.3% Trypan Blue dye, and the stained cells were observed under a microscope. Each data point represents the average of the observations from the four fields of view.

### Enzyme-linked immunosorbent assay (ELISA)

Serum levels of IL-1β were determined using commercial ELISA kits (MB-2899A) according to the manufacturer’s instructions (Meibiao Biotechnology, China). Each ELISA was performed in triplicate. To quantify IL-1β levels, the absorbance of the samples was measured at 490 and 680 nm using a BioTek Epoch microplate spectrophotometer (BioTek Instruments, WinooskiGrödig, USA). Cytokine concentrations are expressed as pg/mgserum protein.

### Statistical analysis

Each experiment was performed at least in triplicates. The results are displayed as the mean ± standard deviation using GraphPad Prism. Statistical significance was determined using one-way ANOVA, with * and ** representing significant (*p* < 0.05) and extremely significant differences (*p* < 0.01), respectively.

## Results

### Deletion of tmRNA gene affects gene expression related to flagellar motility, biofilm formation and amino acid metabolism

**Figure 1.**
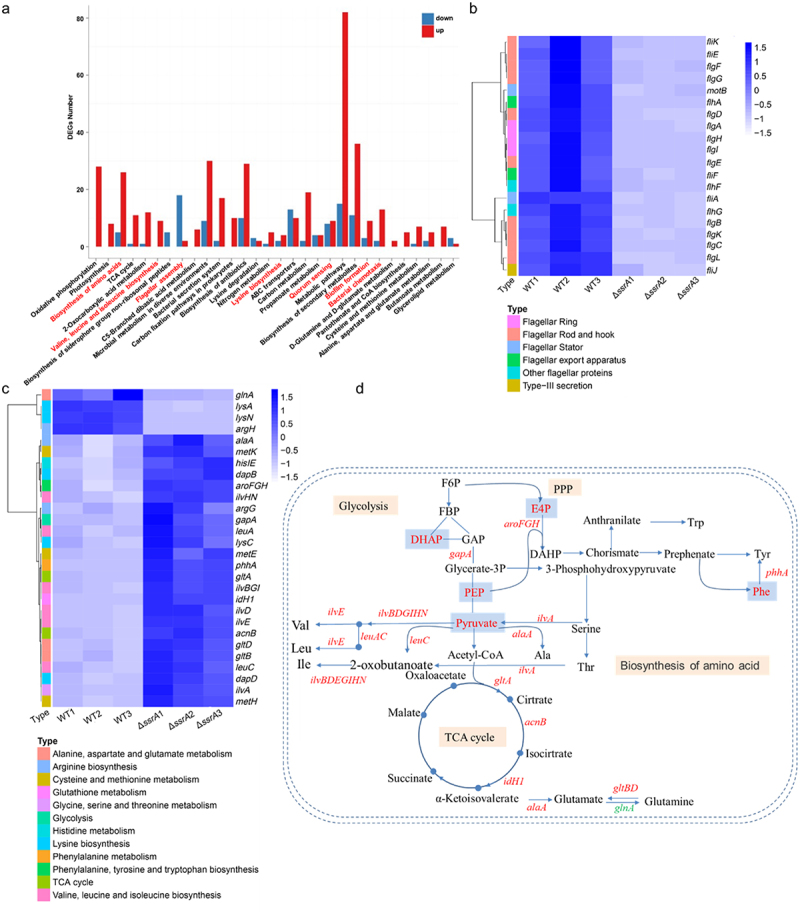
RNA-Seq analysis of WT and Δ*tmRNA*. (a) DEGs number of the most enriched pathway. DEGs: differentially expressed genes. The red and green represented significantly up-regulated and down-regulated genes (log2 fold change>1, *p* value< 0.05) in Δ*tmRNA* strain compared to the wild-type, respectively. (b) The heat map of DEGs associated with flagellum assembly using transcriptome analysis. A comparison was performed between WT and Δ*tmRNA* strain. (c) Heat map illustrating differentially expressed signal pathways associated with amino acid synthesis using transcriptome analysis between WT and Δ*tmRNA* strain. (d) Differential expressions of genes and metabolites involved in amino acid synthesis between WT and Δ*tmRNA* strain. The red and green characters represented significantly up-regulated and down-regulated genes (log2 fold change>1, *p* value< 0.05) in Δ*tmRNA* strain compared to the wild-type, respectively.

To identify the vital regulation pathway of tmRNA, RNA-Seq was used to perform transcriptome analysis of WT and Δ*tmRNA* strains. The top 30 enriched metabolic pathways were identified by KEGG functional annotation and enrichment analysis of DEGs (differentially expressed genes) (Figure 1(a)), including oxidative phosphorylation and amino acid biosynthesis, TCA cycle, flagellar assembly, quorum sensing, biofilm formation and bacterial chemotaxis. Most genes involved in amino acid biosynthesis were significantly upregulated, while numerous downregulated genes were enriched in the flagellar assembly pathway, including the flagellar assembly-related genes *fliE fliF*, *motB* (Figure 1(b)). Furthermore, alterations in transcriptional pathways involved in amino acid synthesis were analyzed after tmRNA knockout, encompassing pathways such as glycolysis, phenylalanine, alanine, valine, leucine, isoleucine, tyrosine and glutamate synthesis (Figure 1(c)). Metabolome data were analyzed to identify the differential metabolites involved in amino acid synthesis after tmRNA knockout, including pyruvate, phosphoenolpyruvate (PEP), dihydroxyacetone phosphate (Glycerone-P), erythrose-4 phosphate (Erythrose-4), phenlalanine and D-ribulose-5 (Figure 1(d)). By combined transcriptome and metabolome data, a large number of differentially expressed genes and metabolites were identified (Figure 1(d)). These results suggest that tmRNA plays a crucial role in flagellar motility, biofilm formation and amino acid metabolism.

### AsfR interacts with tmRNA promoter

**Figure 2.**
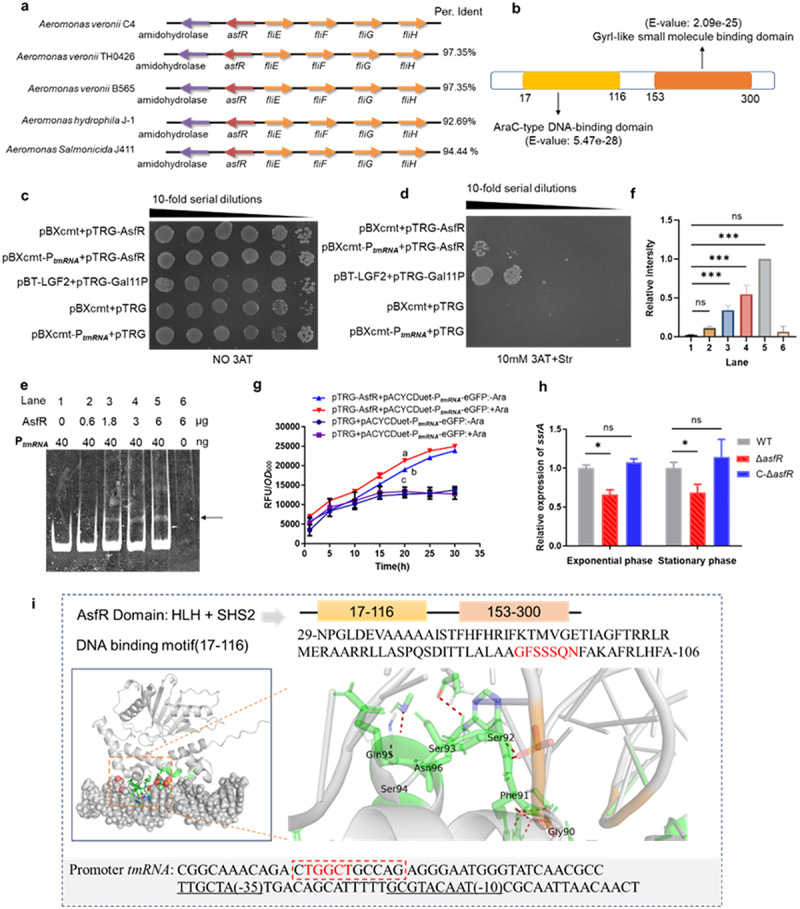
AsfR binding with the promoter region of tmRNA gene encoding tmRNA *in vitro*. (a) Genomic localization of *asfR* and related genes in *Aeromonas* strains. The arrows indicated the direction of gene transcription. (b) Functional domains of AsfR protein predicted by NCBI CD-search. (c) and (d) Bacterial one-hybrid assays grown on the medium in the absence or presence of 10 mM 3-AT. P_*tmRNA*_ represented the promoter of tmRNA gene. (e) EMSA assay of AsfR protein interaction with the tmRNA promoter. The tmRNA promoter and AsfR protein were incubated in EMSA buffer (20 mM HEPES, 100 mM Nacl, 1mM DTT, pH7.4). (f) EMSA quantitative analysis of tmRNA–AsfR interactions through ImageJ. The data was presented as mean ± SD using one-way ANOVA analysis with *** representing significant difference (*P*< 0.001). The EMSA shift signal was enhanced with increasing concentrations of AsfR. (g) Assessment of eGFP fluorescence levels. The strains were transformed with reporter gene alone or with both reporter gene and AsfR. Fluorescence measurements were taken with and without arabinose induction. Ara: arabinose. Significant differences are indicated by different letters. (*p*<·0.05). (h) Quantitative PCR (qPCR) analysis of tmRNA expression during exponential and stationary growth phases. WT: wild type, Δ*asfR*: *asfR* knockout strain, C-*asfR*: complementing *asfR* to Δ*asfR*. Statistical significance was determined using one-way ANOVA, with significance indicated by *p* < 0.05 (*p<·0.05). ns represents no significance. Three independent biological repeats. (i) Molecular docking of AsfR and promoter DNA by AlphaFold3 and HADDOCK. The red-labeled characters indicate the predicted interaction sites, while the red-dotted box marks the conserved palindromic sequence in the promoter region.

To search for upstream transcriptional regulators of tmRNA, we used two prediction tools: MEME and prodoric [27,28]. The transcription factor AsfR (AraC superfamily regulator) was predicted to bind to the promoter of *ssrA* gene, which encode the tmRNA. This prediction was based on the analysis of multiple transcription factors associated with the promoter region (Supplementary Data 1 and 2). In *A. veroni* C4, AsfR exhibited approximately 97% amino acid sequence identity with the homologous proteins in *A. veroni* TH0426 and B565, and about 93% and 94% identity with those of *A. hydrophila* J-1 and *A. salmonicida* J411 respectively. Additionally, the location of *asfR* in these strains remained highly consistent (Figure 2(a)), indicating a high degree of conservation of the *asfR* gene in certain strains of the *Aeromonas* genus. The AsfR protein comprises numerous helical structures, including a tandem of N-terminal AraC family DNA-binding domain and a C-terminal GyrI-like small-molecule binding domain (Figure 2(b)). The N-terminus of AsfR preferentially binds to the target gene, whereas the C-terminus is regulated by small molecules in the environment.

Subsequently, the bacterial one-hybrid system was used to identify the interaction between AsfR and tmRNA promoter. While all tested strains exhibited robust growth without 3-AT (Figure 2(c)), only those harboring both pBXcmt-P_***tmRNA***_ and pTRG-AsfR sustained growth in a medium containing 10 mM 3-AT and streptomycin following serial dilution (Figure 2(d)). The growth pattern resembled that of the positive control, carrying both pBT-LGF2 and pTRG-Gal11^p^. Similar results were obtained in medium containing 2.5 mM, 5 mM and 12 mM 3-AT (Fig. S1). These results suggest that the transcription factor AsfR binds to the tmRNA promoter region. Furthermore, EMSA was used to verify the interaction between AsfR and tmRNA promoter. The AsfR protein (34kDa) was expressed and purified using a Ni-IDA column (Fig. S2). The purified protein was incubated with the 6-FAM labeled tmRNA promoter and analyzed by PAGE. The results revealed that the complex formed by AsfR and the tmRNA promoter caused retardation in the migration of the probe, with AsfR protein (Figure 2(e,f)). Increasing concentrations of AsfR enhanced the EMSA shift signal, indicating a specific DNA – protein interaction. These findings provide evidences that AsfR binds specifically to the tmRNA promoter *in vitro*.

### AsfR positively regulates tmRNA gene

The fluorescence plasmid pACYCDuet-P_***tmRNA***_-eGFP was constructed by integrating the tmRNA promoter with the coding sequence of enhanced green fluorescent protein (EGFP). Co-expression of pTRG-AsfR with pACYCDuet-P_*tmRNA*_-eGFP in *E. coli* resulted in a significant increase in fluorescence intensity at 20 h, demonstrating an extremely significant difference compared with conditions lacking AsfR expression (Figure 2(g)). Both the *asfR-tmRNA* deletion and complemented strains were demonstrated by the colony PCR (Fig S3). In Δ*asfR* strain, the transcription of tmRNA was significantly reduced compared to that in the WT. However, complementation with *asfR* restored tmRNA transcription levels during both the exponential and stationary growth phases (Figure 2(h)), indicating that AsfR exerted a positive regulatory influence on tmRNA expression. Using both AlphaFold 3 and HADDOCK prediction approaches [42,43], we preliminarily identified molecular docking structure and their interaction sites (Figure 2(i)), suggesting that the amino acid residue (GFSSSQN) in AsfR and the sequence (TGGCT) in the tmRNA promoter may play crucial roles in this interaction.

### AsfR affects flagellar motility and biofilm formation by regulating tmRNA

**Figure 3.**
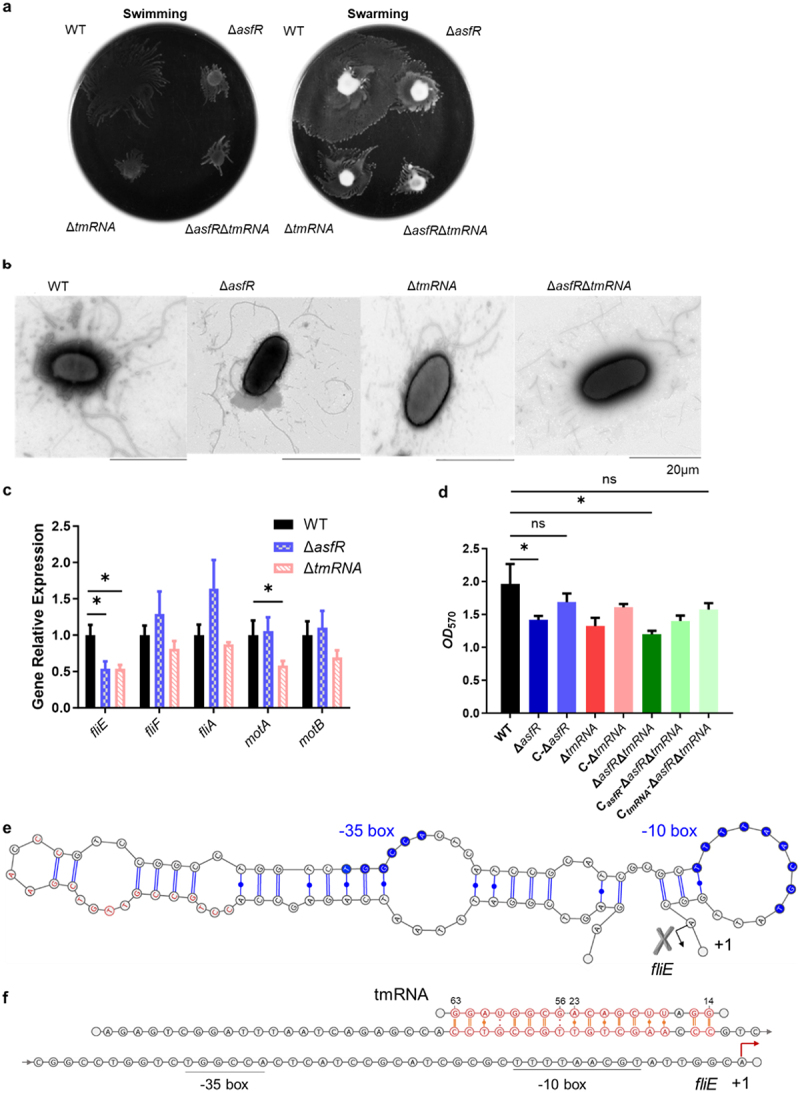
AsfR affects the motility of flagella and biofilm formation by regulating tmRNA expression. (a) Swimming and swarming assays. (b) Flagellar structure observation by transmission electron microscope (TEM). Scale bar, 20μm. (c) The qPCR analysis of genes related to flagellum synthesis. (d) Determination of biofilm formation. C-Δ*asfR*: Complemented *asfR* to Δ*asfR* strain, C-Δ*tmRNA*: Complemented *tmRNA*: to Δ*tmRNA*: strain, C_*asfR*_-Δ*asfR*Δ*tmRNA*: Complemented *asfR* to Δ*asfR*Δ*tmRNA*: strain, C_*tmRNA*_-Δ*asfR*Δ*tmRNA*: Complemented *tmRNA* to Δ*asfR*Δ*tmRNA*. One-way ANOVA was used for statistical analysis (*p<·0.05), three independent biological repeats. (e) Predicted stem – loop structure of *fliE* promoter. The centroid secondary structure of the promoter was predicted using RNAfold [49], rendered with VARNA (Java) [50]. The *fliE* promoter sites interacting with tmRNA, identified by IntaRNA [51], are highlighted in red. (f) Predicted base-pairing model between tmRNA and the *fliE* promoter. Complementary base pairs are highlighted in red. The transcriptional start site is indicated by an arrow.

Transcriptome data revealed the down-regulation of flagellar genes, including *fliE*, *fliF*, *fliA*, *motA* and *motB* in the tmRNA deletion strain (Figure 1(b)). To investigate whether the effect of AsfR on flagellar formation was mediated by tmRNA regulation, swimming and swarming assays were performed and the results demonstrated weaker motility in the deletion strains of *asfR* and *tmRNA* (Figure 3(a)). In agreement with previous studies [47], the wild-type strains possessed a single polar flagellum along with multiple lateral flagella by TEM imaging, whereas the mutant strains were restricted to a single flagellum (Figure 3(b)), indicating a loss of flagella formation. The qPCR analysis further demonstrated a significant downregulation of the flagellum gene *fliE* in the deletion strains of *asfR* and *tmRNA* compared to the WT strain (Figure 3(c)). Collectively, these results suggest that AsfR regulates tmRNA, thereby influencing the synthesis of flagella and motility of bacteria. Considering the essential role of flagellar motility in biofilm formation [48], we evaluated biofilm formations of *A. veronii* derivatives. The amount of biofilm was significantly decreased in Δ*asfR*、Δ*tmRNA*、Δ*asfR*Δ*tmRNA* strains compared to the WT strain, while the biofilm-forming capacities of the strains were restored after complementation with *asfR* and *tmRNA* genes (Figure 3(d)).

To investigate direct regulatory interactions, we analyzed the base complementarity of the tmRNA with promoters of flagella-related genes including *fliE*, *fliF*, *fliA*, *motA* and *motB*. Regions of complementarity are highlighted in blue boxes (Fig. S4). Further analysis revealed that the promoter region of *fliE* forms the stem-loop structure, making it difficult for RNA polymerase to bind, resulting in a low transcription level of *fliE* (Figure 3(e)). We hypothesized that after tmRNA interacts with the *fliE* promoter, the sites are exposed, facilitating the binding of RNA polymerase and increasing the transcription level of *fliE* (Figure 3(f)). Similar regulatory mechanisms have been reported that Goodson et al. identified small RNA RTA-3 that activate transcription by promoting RNA polymerase engagement at the promoter [52]. These findings suggest that AsfR modulates the motility and biofilm formation by regulating tmRNA expression in *A. veronii*.

### Deletion of asfR and tmRNA causes growth defects in oxidative stress and virulence attenuation of *A. veronii*

**Figure 4.**
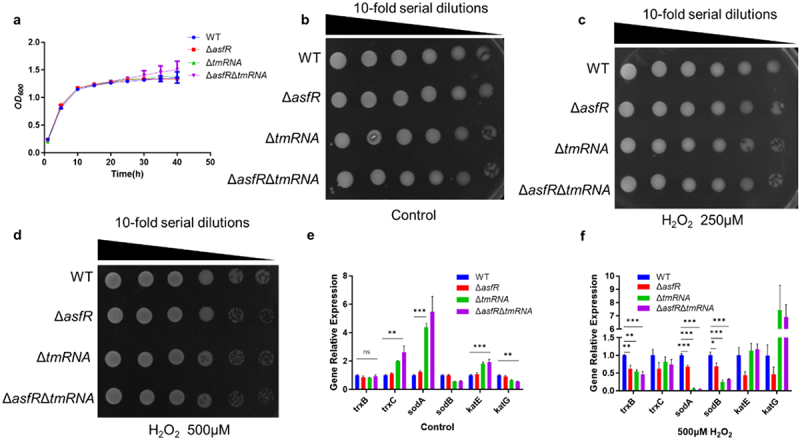
Growth assessments in Δ*asfR*Δ*tmRNA* strain under various culture conditions. (a) Bacterial growth under nutrient-rich conditions (LB medium). (b) Bacterial growth under nutrient-deprived conditions (M9 medium). (c) Bacterial growth under the treatment of 250μM H_2_O_2_ (M9 medium). (d) Bacterial growth under the treatment of 500μM H_2_O_2_ (M9 medium) (e) RT-qPCR assay of antioxidant gene in absence of H_2_O_2_. (f) RT-qPCR assay of antioxidant gene with 500μM H_2_O_2_ treatment. One-way ANOVA was used for statistical analysis (*p<·0.05, **p<·0.01, ***p<·0.001), three independent biological repeats.

Oxidative stress has been shown to enhance the efficacy of certain drugs [53]. To identify the effect of AsfR and tmRNA on growth and metabolism, oxidative stress analyses were performed in M9 medium with different concentrations of H_2_O_2_. No statistically significant differences in growth were observed between the WT and mutant strains under nutrient-rich conditions (LB) and nutrient-deprived (M9) conditions (Figure 4(a,b)). However, conspicuous differences between the WT and Δ*asfR*Δ*tmRNA* strains were observed under oxidative stress conditions (M9 medium) (Figure 4(c) and Fig. S5); In particular, the differences between WT and mutants were extremely significant when treatment with 500 μM H_2_O_2_ (Figure 4(d)). To clarify potential mechanism of reduced resistance in Δ*asfR*/Δ*tmRNA* mutants, we performed RT-qPCR assay of antioxidant gene including *trxB* (Trx reductase), *trxC*, *sodA* (superoxide dismutases), *sodB*, *katG* (catalase G) and *katE*, in WT and mutants. The results showed negligible difference of *trxB* expression between WT and mutant strains without H_2_O_2_ treatment (Figure 4(e)). Following H_2_O_2_ treatment, the expression of *trxB*, *sodA*, and *sodB* was strikingly reduced in the mutants compared with WT (Figure 4(f)), suggesting that gene knockout of *asfR* and *tmRNA* impaired the oxidative stress response by limiting antioxidant gene expression. Furthermore, in the absence of H_2_O_2_, knockout strains showed increased expression of *trxC, katE*, and *sodA*, suggesting that disruption of certain antioxidant genes triggered compensatory upregulation of others (Figure 4(e)). These findings indicate that the regulatory pathways of AsfR and tmRNA are essential for maintaining antioxidant capacity.

**Figure 5.**
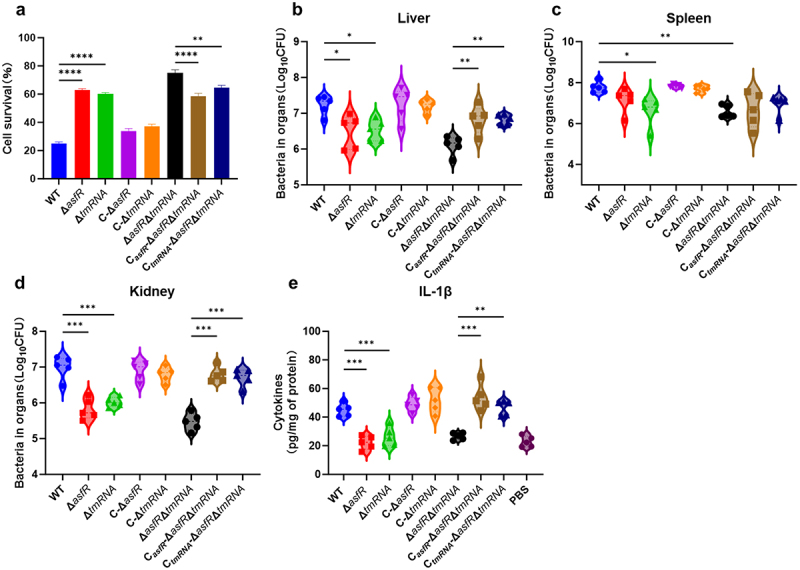
Assessment of virulence in the mutant strains. (a) Evaluation of fibroblast L929 cell viability after infection with *A. veronii* derivatives. Experimental data were collected from three independent biological replicates. (b-d) Analysis of tissue colonization using mouse model. The livers, spleens, and kidneys were harvested to enumerate bacterial recovery after post-infection. Each experiment was performed in five biological repeats. (e): Levels of IL-Iβ following infection. One-way ANOVA was used for statistical analysis (*p<·0.05, **p<·0.01, ***p<·0.001).

To investigate the pathogenicity of the mutant strains of *A. veronii*, a fibroblast cell line (L929) based co-culture model was designed. Compared to the WT and mutant strains, the deletion strains of *asfR* and *tmRNA* exhibited reduced pathogenicity and resulted in a higher survival rate of L929 cells (Figure 5(a)). Additionally, to evaluate the role of AsfR and tmRNA in *A. veronii* virulence in a mouse model, doses of up to 6 × 10^6^ CFU were administered by intraperitoneal injection. The virulence of Δ*asfR*, Δ*tmRNA*, and Δ*asfR*Δ*tmRNA* was significantly reduced in mouse liver, spleen, and kidney infections compared with the WT (Figure 5(b-d)). Conversely, the virulence defect of tmRNA mutants was compensated for by complementation with *tmRNA*, and that in *asfR* mutant was restored upon complementation with *asfR* (Figure 5(b-d)). Compared with WT, Δ*asfR*, Δ*tmRNA*, Δ*asfR*Δ*tmRNA* mutants did not cause significant infection in the mouse heart and lung (Fig. S6). Microorganisms recognized as the invading by pathogen-associated molecular patterns (PAMPs) trigger host immune responses, such as IL-1β secretion [54]. Remarkably, the secretion of IL-1β in monocytes cells stimulated by PAMPs displayed significant differences (Figure 5(e)). Deletion of *asfR* and *tmRNA* results in lower secretion of cytokine IL-1β during mouse infection. We speculated that the *asfR* and *tmRNA* mutants possessed weaker flagella motility and host invasion ability, leading to a weaker pro-inflammatory response. These data demonstrate the essential role of a functional AsfR-tmRNA mediated pathway in the virulence and pathogenesis of *A. veronii*.

## Discussion

Starting from the screening of upstream transcription factors regulating tmRNA expression, we investigated into the regulatory mechanism of AsfR and tmRNA for flagellum function and virulence. The promoter region of tmRNA interacted with AsfR, which is composed of both the AraC family DNA-binding domain at the N-terminus and a GyrI-like domain at the C-terminus (Figure 2(b)). The GyrI-like domain is characterized by a SHS2 (strand-helix-strand-strand) module that binds small molecules [55] and participates in transcriptional activation and DNA gyrase inhibition. In particular, the structure of AsfR was distinguished from the conventional AraC superfamily which exhibited a tandem of DNA-binding and ligand-binding domains, positioned orderly at the C- and *N*- termini [56,57].

In canonical AraC family regulators such as ToxT, the N-terminal ligand-binding domain modulates the activity of the C-terminal DNA-binding domain, enabling effector molecules (fatty acids) to function as allosteric caps that suppress DNA binding or transcriptional activation [58,59]. By contrast, AsfR exhibits an inverted domain arrangement, with the DNA-binding domain at the N-terminus and the ligand-binding domain at the C-terminus. This reversal suggests a fundamentally different regulatory mechanism: DNA recognition may occur prior to effector sensing, with the C-terminal ligand-binding domain acting as a secondary regulatory module that modulates transcription after DNA engagement. Such a mechanism could allow AsfR to establish promoter occupancy independent of effector availability, while retaining the capacity for effector-dependent fine-tuning. The domain inversion therefore implies a shift from effector-gated DNA binding, typical of canonical AraC regulators, to effector-modulated activity on an already bound DNA – protein complex. The three-dimensional structure of the AsfR protein will need be further verified by Χ ray crystal diffraction or nuclear magnetic resonance (NMR).

The AraC family protein from the nat operon was reported to promote the expression of N-acetyltransferase (NAT) gene clusters associated with cholesterol degradation [60]. In this study, AsfR was identified as a transcriptional regulator that upregulated the transcriptional expression of tmRNA, thereby enhancing the expression of the flagellar gene *fliE* (Figure 3(c)). The flagella III secretory system in bacteria plays multiple roles in the secretion of virulence effector factors, bacterial motility, biofilm formation and environmental signal sensing [22,61]. FliE constitutes the proximal rod of the flagellar basal body, and mutation of the *fliE* gene causes poor movement of flagella in *S. enterica* [62]. The deletion of *asfR* or *tmRNA* resulted in reduced flagellar motility (Figure 3(a)), with fewer flagella observed on the bacterial surface in the knockout strains than in the WT (Figure 3(b)).

**Figure 6.**
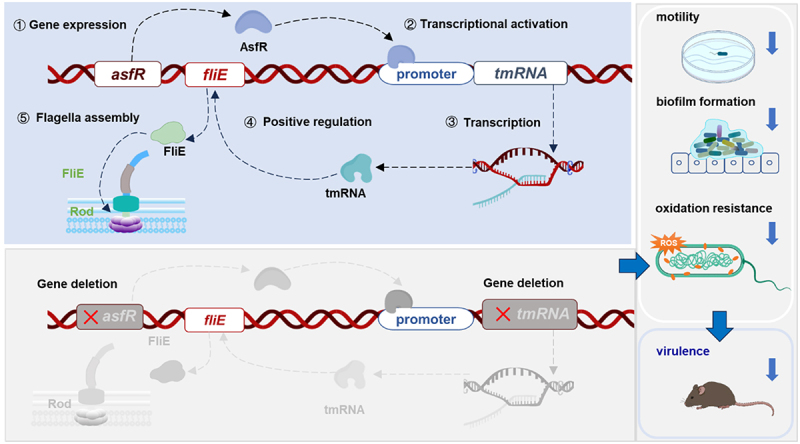
The proposed model of the effects of AsfR and tmRNA on motility, virulence in *A. veronii*. The transcriptional factor AsfR promotes the transcription of tmRNA gene. Mature tmRNA, through an unknown mechanism, positively regulates the expression of the flagellar gene *fliE*, facilitating flagellar assembly. Flagella enable bacterial motility and contribute to biofilm formation, which aids in resisting adverse environmental conditions. Additionally, the deletion of *asfR* and *tmRNA* influences the antioxidant capacity and pathogenicity. Collectively, the AsfR/tmRNA axis regulates flagellar motility, biofilm formation and antioxidant capacity, all of which contribute to the virulence of *A. veronii*.

Flagellar movement significantly influences biofilm formation, as it facilitates bacterial attachment to solid surfaces through motility and physical adsorption of the flagella [63]. Impaired flagellar movement reduces biofilm formation in *Listeria monocytogenes* and *Aeromonas caviae* [23,39]. However, in *Pseudomonas aeruginosa*, loss of flagellar movement increases biofilm formation in *sadB* knockout, suggesting that flagellar motility may not be the decisive factor in biofilm formation [64]. Our study revealed that AsfR mediated biofilm formation in *A. veronii* C4 by positively regulating tmRNA (Figure 3(d)). Given the intimate association between the flagellar type III secretion system and virulence mechanisms of pathogens, dysfunction of the AsfR-tmRNA pathway resulted in decreased biofilm formation, reduced infection in mouse tissue, and attenuated host immune responses (Figure 6). Strikingly, deletion of *asfR* and *tmRNA* resulted in lower virulence and secretion of cytokine IL-1β during mouse infection (Figure 5).

Small non-coding RNAs (sRNAs) are involved in metabolic processes, cancer progression, and immune regulation [65]. For instance, the sRNA GcvB inhibits the transcription of genes such as *gltI* and *argT* through complementary base pairing with mRNAs, thereby hindering amino acid uptake and utilization [66]. The sRNA SR1 detects structural alterations downstream of the *ahrC* ribosomal binding site, inhibiting gene translation and regulating arginine metabolism [40]. tmRNA, a unique non-coding RNA, is widely conserved across bacteria, bacteriophages, and archaea. However, it’s biological functions remain largely unexplored [67]. Oxadiazoles have been reported to interrupt the biological function of tmRNA, inhibiting the growth of various pathogens, including *Shigella flexneri*, *Bacillus anthracis*, *Mycolicibacterium smegmatis*, *Francisella tularensis* [19,68]. In this study, we found that tmRNA deletion affected amino acid metabolism and flagellar assembly pathways, which may underlie the reduced virulence and antioxidant capacity observed in AsfR and tmRNA deletion strains (Figure 1(c,d) and 6). Compared to the WT and mutant strains, the deletion strains of *asfR* and *tmRNA* exhibited reduced pathogenicity in L929 cells and in a mouse model. Exploring the precise regulatory roles of AsfR and tmRNA pathways is of great significance for elucidating the mechanism of bacterial resistance.

In summary, this study identified AsfR as a positive regulator of tmRNA expression and investigated the effects of the AsfR-tmRNA axis on flagella motility and biofilm formation. Our findings revealed a decrease in the transcriptional expression of the flagellar gene *fliE* upon deletion of both *asfR* and *tmRNA*, which underlie the impaired motility and diminished biofilm formation observed in these mutants. Furthermore, deletions of both AsfR and tmRNA reduced antioxidant capacity and virulence of *A. veronii*. These findings underscore the essential roles of AsfR and tmRNA in flagellar motility and bacterial virulence, providing insights that may pave the way for the treatment of bacterial diseases.

## Supplementary Material

20251015 SI supplement information.docx

Supplementary data 1 and 2.xlsx

Figures.zip

## Data Availability

All datasets that support this study are included in the article/Supplementary Material. The Data and supplementary files are available in figshare at https://doi.org/10.6084/m9.figshare.28816460.v3 [69]. The transcriptome data were deposited in the NCBI database under accession number GSE120603 (https://www.ncbi.nlm.nih.gov/geo/query/acc.cgi?acc=GSE120603). The metabolome data were available in the Metabolights repository (accession code: MTBLS1191; https://www.ebi.ac.uk/metabolights/). These datasets were identical to those used in our original publication [38], but have undergone additional advanced computational analysis.
