# Supplementary material for: The regulation of AsfR on tmRNA expression mediates bacterial motility and virulence in Aeromonas veronii
Source: Virulence. 2025 Dec 10;17(1):2602247. doi: 10.1080/21505594.2025.2602247 (PMC12710916; doi:10.1080/21505594.2025.2602247)
Supplement: 20251015 SI supplement information.docx [file KVIR_A_2602247_SM0849.docx]

## **Supplementary Figure**


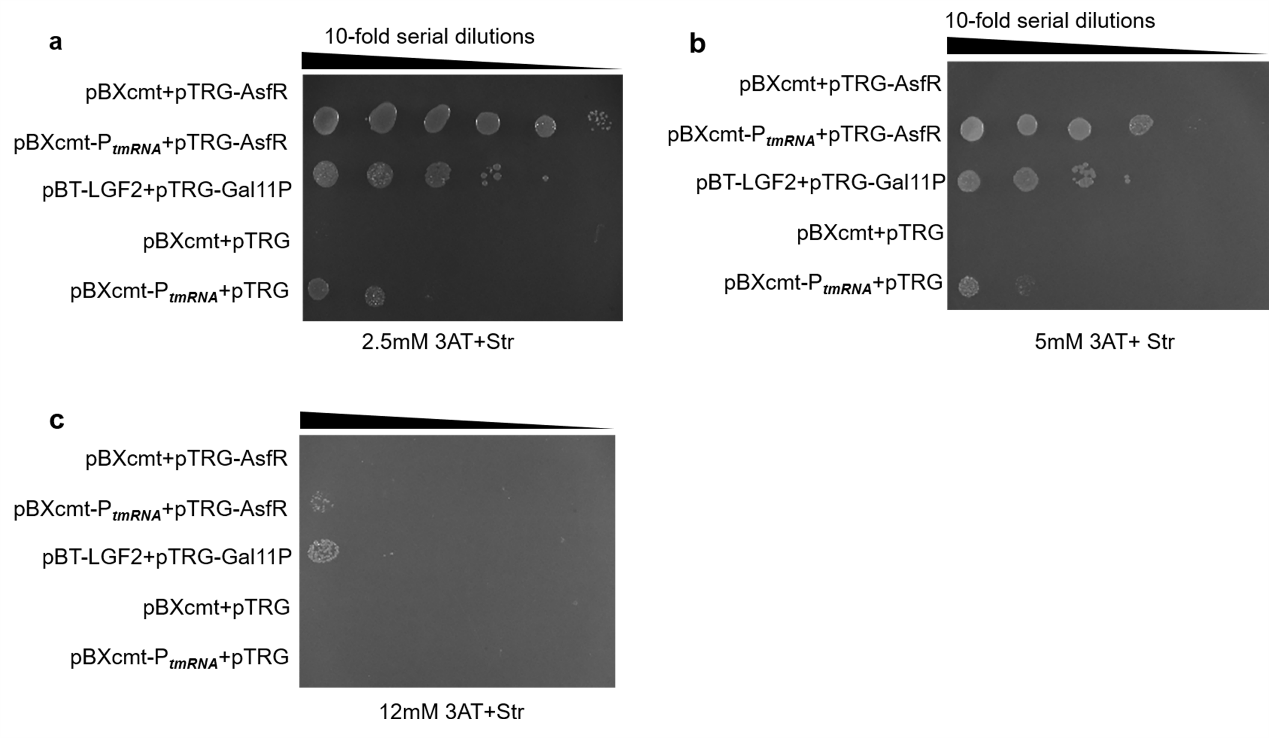


Supplementary Figure S1 Bacterial one-hybrid assays on the medium in the presence of 3-AT.

The different concentration of 3-AT is 2.5 mM, 5 mM or 12mM. Str: streptomycin. P***_tmRNA_*** represented the tmRNA promoter encoded by *ssrA*.

**
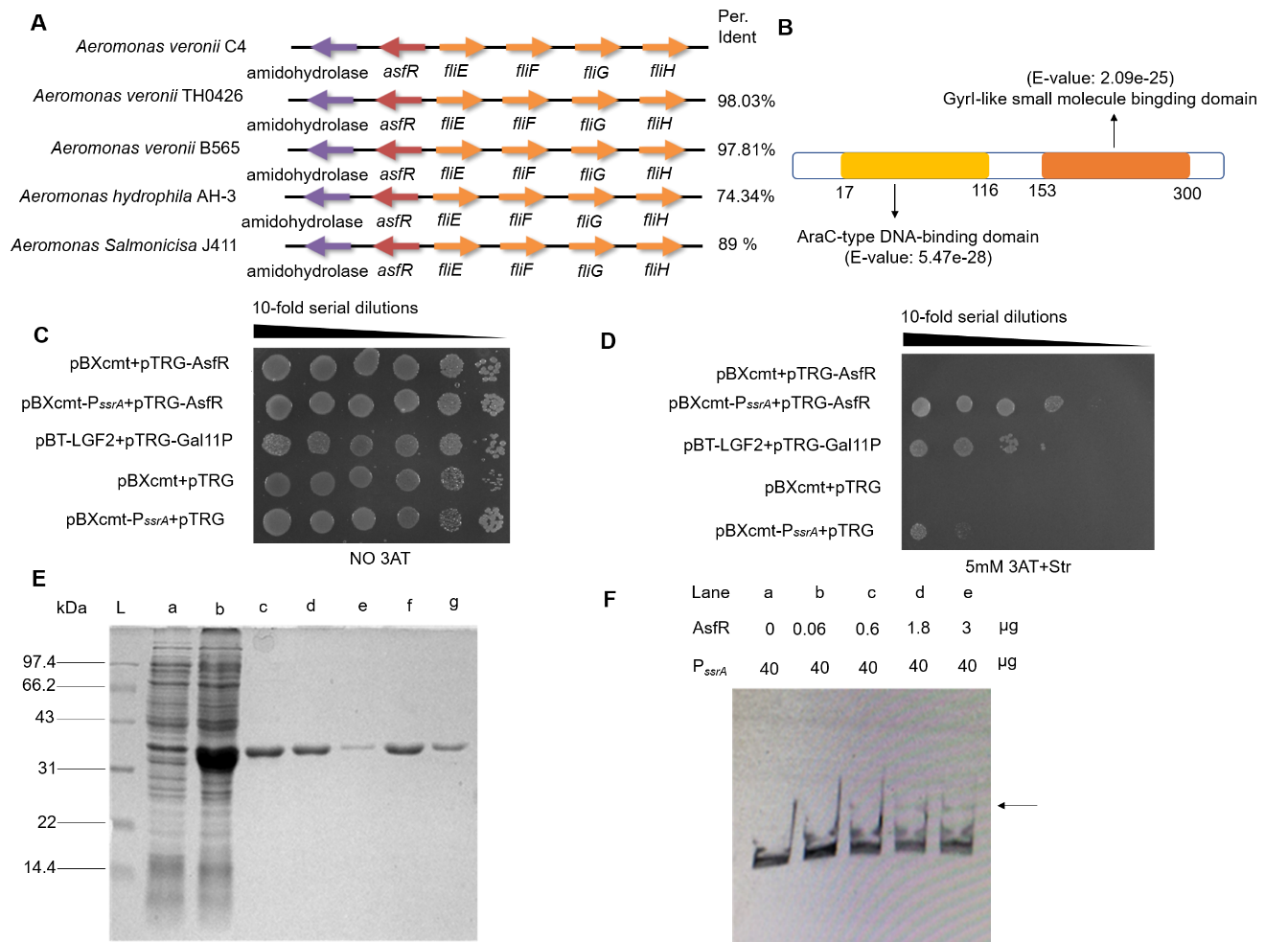
**

Supplementary Figure S2 SDS-PAGE of AsfR Purification

Lane L, protein marker. Lanes a-b, supernatant lysates before and after IPTG induction. Lanes c-e, elution with 250 mM imidazole. Lanes f-g, proteins after dialysis.


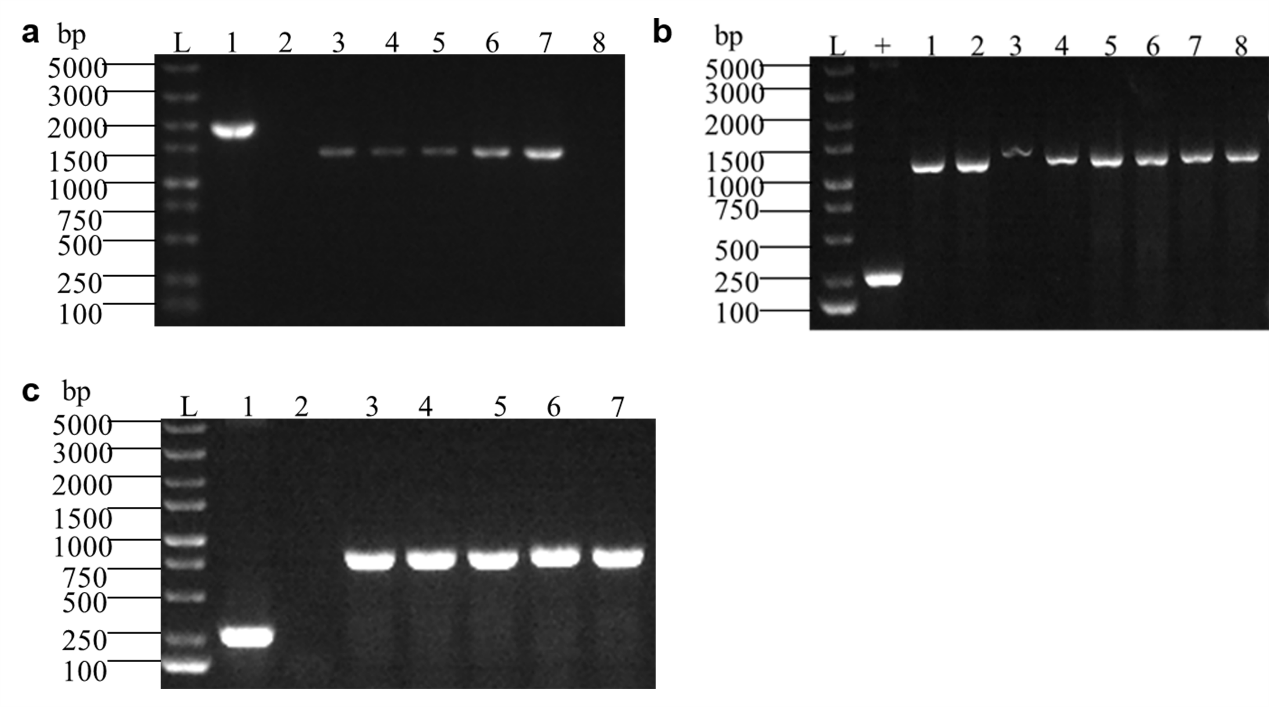


Supplementary Figure S3 Colony PCR

(a) Identification of the *asfR* gene deletion for the strain Δ*asfR*Δ*tmRNA*. L*:* DL5000 DNA Marker; 1: The positive control; 2: The negative control; 3~8: The PCR products. The PCR amplifications were performed using target gene specific primers F0/R0. The sequence of the *asfR* homologous arm is approximately 1500 bp, which is shorter than the amplified length in the wild-type. (b) Identification of the complement strains of *asfR* gene. 1: The positive control; 1~4: The PCR products of the candidate transformants of C-Δ*asfR*; 5~8: The PCR products of the candidate transformants of C***_asfR_***-Δ*asfR*Δ*tmRNA*. (c) Identification of the complement strains of *tmRNA* gene. 1: The positive control; 2: The negative control; 3~7: The PCR products of the candidate transformants of C***_tmRNA_***-Δ*asfR*Δ*tmRNA*.


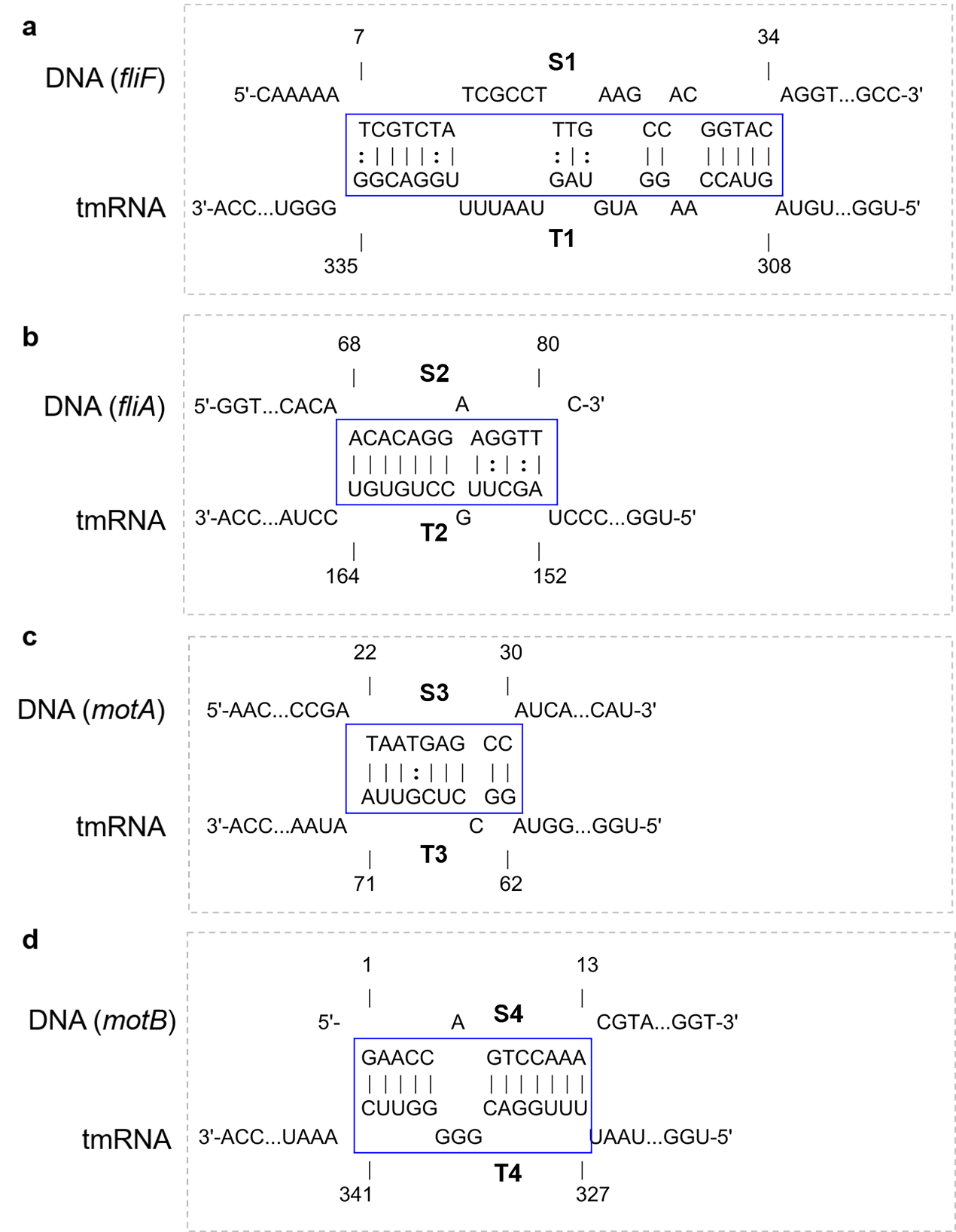


Supplementary Figure S4 Base complementarity analysis between tmRNA and the promoters of flagella-related genes.

The interaction sites between tmRNA and the promoters of *fliF*, *fliA*, *motA*, and *motB* genes were predicted through IntaRNA software [1]. Blue boxes denote complementary base-pairing regions.


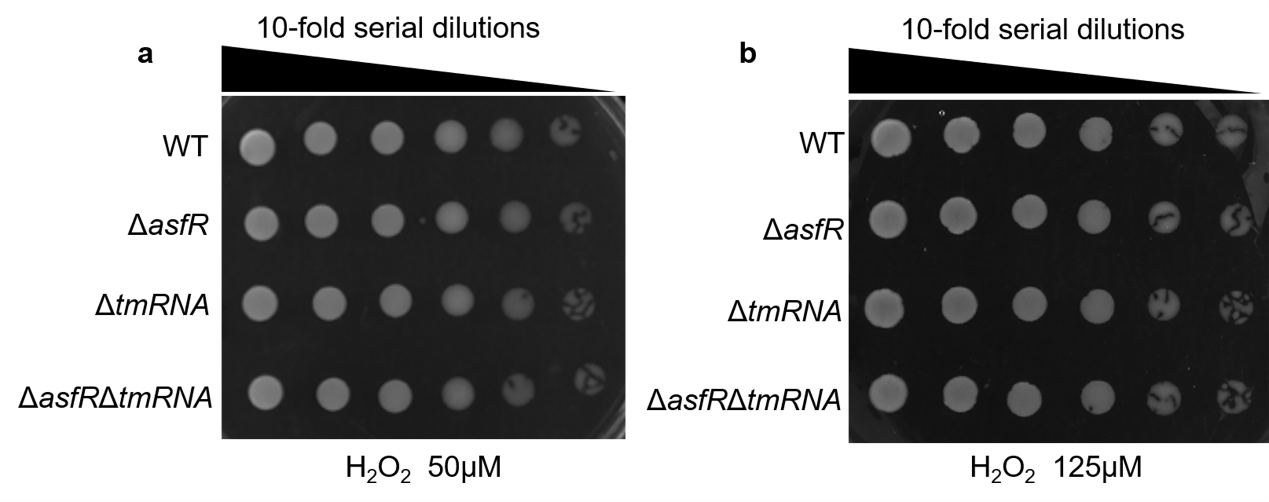


Supplementary Figure S5 Bacterial growth inhibition by H_2_O_2_


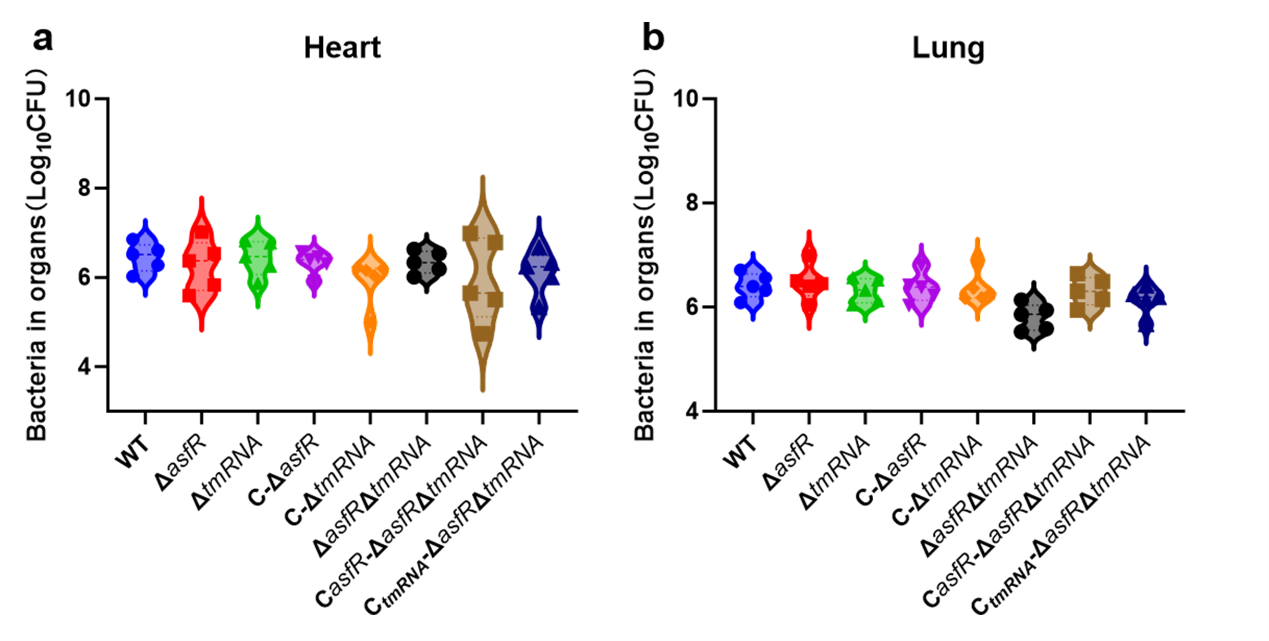


Supplementary Figure S6 Analysis of tissue colonization on mouse model for virulence assessment. The hearts and lungs were harvested to enumerate bacterial recovery at post-infection. Each experiment was performed in five biological repeats.

## **S**upplementary Table

**Supplementary Table S1 Bacterial strains used in this study**

| Strains | Description | Sources |
| --- | --- | --- |
| *Aeromonas veronii* C4 | Wild type, ampicillin resistance | [2] |
| *A. veronii* Δ*asfR* | Ampicillin resistance, *asfR* gene was deleted in *A. veronii* C4 | [3] |
| *A. veronii* C-Δ*asfR* | Ampicillin resistance, *asfR* gene was complemented functionally in *A. veronii* Δ*asfR* | This study (Materials and methods) |
| *A. veronii* Δ*tmRNA* | Ampicillin resistance, *ssrA* gene (encoding tmRNA) was deleted in *A. veronii* C4 | [4] |
| *A. veronii* C-Δ*tmRNA* | *tmRNA* gene was complemented functionally in *A. veronii* | This study  (Materials and methods) |
| *A. veronii* Δ*asfR*Δ*tmRNA* | Ampicillin resistance, *asfR* and *tmRNA* gene were deleted in *A. veronii* C4 | This study  (Materials and methods) |
| *A. veronii* C*_asfR_*-Δ*asfR*Δ*tmRNA* | *asfR* gene was complemented functionally in *A. veronii* Δ*asfR*Δ*tmRNA* | This study  (Materials and methods) |
| *A. veronii* C*_tmRNA_*-Δ*asfR*Δ*tmRNA* | *tmRNA* gene was complemented functionally in *A. veronii* Δ*asfR*Δ*tmRNA* | This study  (Materials and methods) |
| *E.coli* XL1-Blue MRF*'* | Kanamycin resistance, Propagates pTRG, pBT, pBX-cmT and relevant derivatives | Keysight Technologies Inc, Santa Clara, CA |
| *E.coli* XL1-Blue MRF'  reporter strain | Kanamycin resistance, Expresses pTRG, pBT, pBX-cmT and relevant derivatives for bacterial one-hybrid systems | Keysight Technologies Inc, Santa Clara, CA |
| *E.coli* WM3064 | Dap (diaminopimelic acid) auxotroph. | [5] |
| *E. coli* BL21 (DE3) | Protein expression host | New England Biolabs, Ipswich, MA |

**Supplementary Table S2 Plasmids used in this study**

| plasmid | Description | Sources |
| --- | --- | --- |
| pRE112 | Suicide plasmid for gene knock out, chloramphenicol resistance, containing *sacB* gene for sucrose selection. | [2] |
| pRE112-Δ*asfR* | pRE112 derivative, containing the upstream and downstream homologous arms of genes | This study  **(**Materials and methods**)** |
| pTRG | The plasmid for Bacterial one-hybrid, tetracycline resistance, ColE1 origin of replication, lpp and lac-UV5 promoter, encoding α-subunit of RNA polymerase | Stratagene, USA |
| pTRG-Gal11p | pTRG derivative, tetracycline resistance, interaction control plasmid encoding a domain of the mutant form of the Gal11 protein | Lab stock |
| pTRG-AsfR | pTRG derivative, tetracycline resistance, contains *asfR* gene | This study  **(**Materials and methods**)** |
| pBT | The plasmid for Bacterial one-hybrid, chloramphenicol resistance, p15A origin, includes HIS3 and *aadA* reporter gene. | Stratagene, USA |
| pBT-LGF2 | pBT derivative, chloramphenicol resistance, interaction control plasmid encoding the dimerization domain of the Gal4 transcriptional activator protein | Stratagene, USA |
| pACYCDuet- P***_tmRNA_*** -eGFP | The plasmid for Enhanced GFP fluorescent assays, chloramphenicol resistance, p15A origin, containing *tmRNA* promoter and eGFP gene. | This study  **(**Materials and methods**)** |
| pBXcmT | The plasmid for Bacterial one-hybrid, chloramphenicol resistance, Ori1 origin, including HIS3-*aadA* reporter gene for | [6] |
| pBXcmT-P***_tmRNA_*** | pBXcmT derivative, chloramphenicol resistance, Ori1 origin, includes HIS3-*aadA* reporter gene, contains *tmRNA* promoter | This study  **(**Materials and methods**)** |
| pBBR-MCS-2 | The plasmid for gene complement, Kanamycin resistance, OriV origin | Lab stock |
| pBBR-AsfR | The plasmid for gene complement of *asfR* gene, Kanamycin resistance | This study  **(**Materials and methods**)** |

**Table S3 Primers used in this study**

| Primer | Prime Sequence(5’-3’) | Description |
| --- | --- | --- |
| *A. veronii* C4 | F: ATGGTCGCAGAGCTTGTC | The primers for specifically confirming *A. veronii* C4 |
|  | R: CAGCACAATAGAACACCAGAC |  |
| pRE112 | F: ACATAGCCCCACTGTTCGT | The primers for specifically confirming pRE112 plasmid |
|  | R: TTTTCGTCTCAGCCAATCC |  |
| *asfR* | F0: GGTGCCCTCATCGTTTATCAG | The primers for specifically confirming *asfR* gene knockout |
|  | R0: AGCTCTACATCGCCCAGTGTC |  |
| *asfR* | F1: CATGAATTCCCGGGAGAGCTCTATT  GGCGTCGCTTATACCG | The primers for amplification of the upstream homologous arm of *asfR* |
|  | R1: CATCGGTGATCCTTGAACACGGGTT  TGCG |  |
|  | F2: GTGTTCAAGGATCACCGATGAGAC  CCGTTG | The primers for amplification of the downstream homologous arm of *asfR* |
|  | R2: CAAGCTTCTTCTAGAGGTACCTTGC  TGCACCTGCTCAACC |  |
| pBBR | F: TGTAAAACGACGGCCAGT | The primers for specifically confirming pBBR plasmid |
|  | R: CAGGAAACAGCTATGAC |  |
| pBBR-*asfR* | F: CGGAATTCTTGCTGGCTGGCAAGGA GTC | The primers for amplification of *asfR* gene to construct pBBR-*asfR* |
|  | R: GCTCTAGATTATCGGTTGATCAGCGG  GATAC |  |
| pTRG | F: TGGCTGAACAACTGGAAGCT | The primers for specifically confirming pTRG plasmid |
|  | R: ATTCGTCGCCCGCCATAA |  |
| pTRG-AsfR | F: CGGGATCCTTGCTGGCTGGCAAGGA  GTC | The primers for amplification of *asfR* gene to construct pTRG-*asfR* |
|  | R: GGAATTCTTATCGGTTGATCAGCGGG |  |
| pACYCDuet- P***_tmRNA_*** -eGFP | F1: CGGGGTACCTACTGGCAGGATCAGGA  CG | The primers for amplification of *tmRNA* gene promoter to construct pACYCDuet- P***_tmRNA_*** -eGFP |
|  | R1: CATGGTATATCTCCTTCTTCCAGTTG  TTAATTGCGATTG |  |
|  | F2: TGGAAGAAGGAGATATACCATGGTG  AGCAAGGGCGAGG | The primers for amplification of eGFP gene to construct pACYCDuet- P***_tmRNA_*** -eGFP |
|  | R2: GGAATTCTTACTTGTACAGCTCGTCC  ATG |  |
| pET-AsfR | F: CCGGAATTCTTGCTGGCTGGCAAGGA  GT | The primers for amplification of *asfR* gene to construct pET-*asfR* |
|  | R: GGGAAGCTTTTATCGGTTGATCAGCG  GGA |  |

**References**

[1] Mann M, Wright PR, Backofen R. IntaRNA 2.0: enhanced and customizable prediction of RNA–RNA interactions. Nucleic Acids Res. 2017;45(W1):W435-W439.

[2] Liu Z, Liu P, Liu S, et al. Small protein B upregulates sensor kinase *bvgS* expression in *Aeromonas veronii*. Front Microbiol. 2015;6:579.

[3] Chang H, Ma X, Li H, et al. Construction and functional identification of *asfR* knockout strain of *Aeromonas veronii* [In Chinese]. Genomics and Applied Biology. 2022;41(05):1037-1047.

[4] Peng M, Cao X, Tang Y, et al. Large-scale identification of trans-translation substrates targeted by tmRNA in *Aeromonas veronii*. Microb Pathog. 2020;145:104226.

[5] Dehio C, Meyer M. Maintenance of broad-host-range incompatibility group P and group Q plasmids and transposition of Tn5 in *Bartonella henselae* following conjugal plasmid transfer from *Escherichia coli*. J Bacteriol. 1997;179(2):538-540.

[6] Guo M, Feng H, Zhang J, et al. Dissecting transcription regulatory pathways through a new bacterial one-hybrid reporter system. Genome Res. 2009;19(7):1301-1308.
