# Supplementary figures and images for: The regulation of AsfR on tmRNA expression mediates bacterial motility and virulence in Aeromonas veronii
Source: Virulence. 2025 Dec 10;17(1):2602247. doi: 10.1080/21505594.2025.2602247 (PMC12710916; doi:10.1080/21505594.2025.2602247)

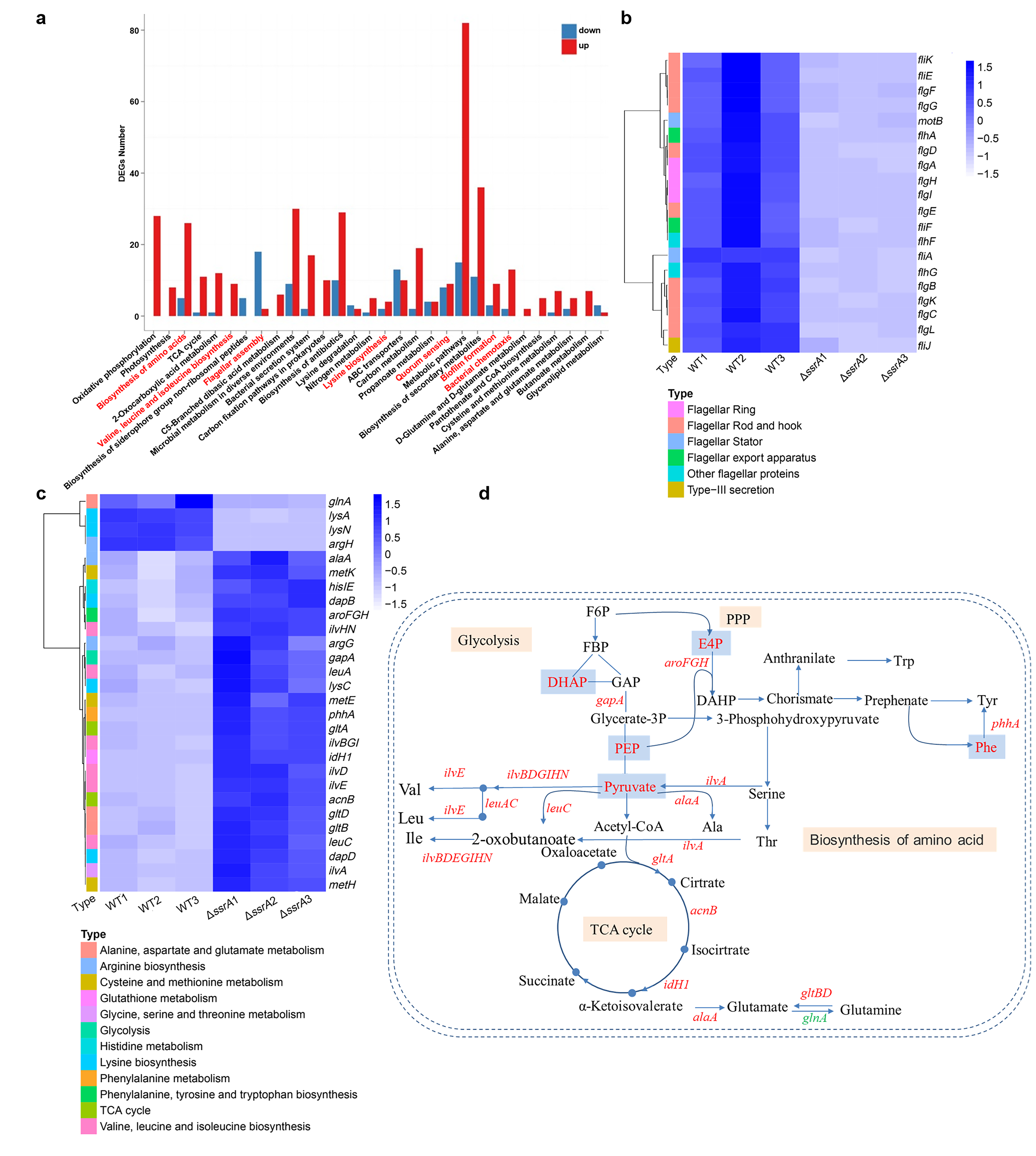

Supplement: Figures.zip [file KVIR_A_2602247_SM0847.zip › Figures/Figure1.tif]

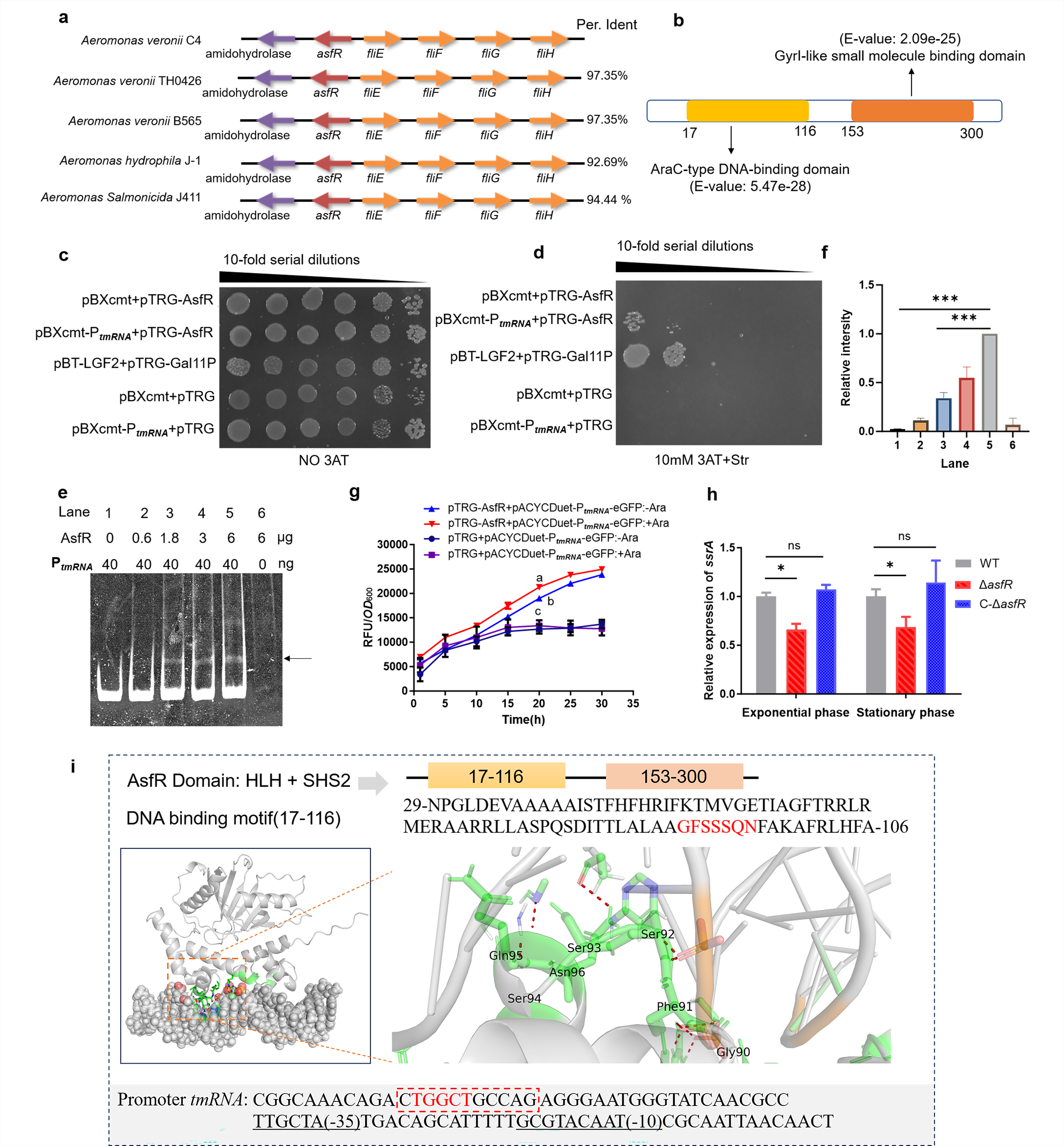

Supplement: Figures.zip [file KVIR_A_2602247_SM0847.zip › Figures/Figure2.tif]

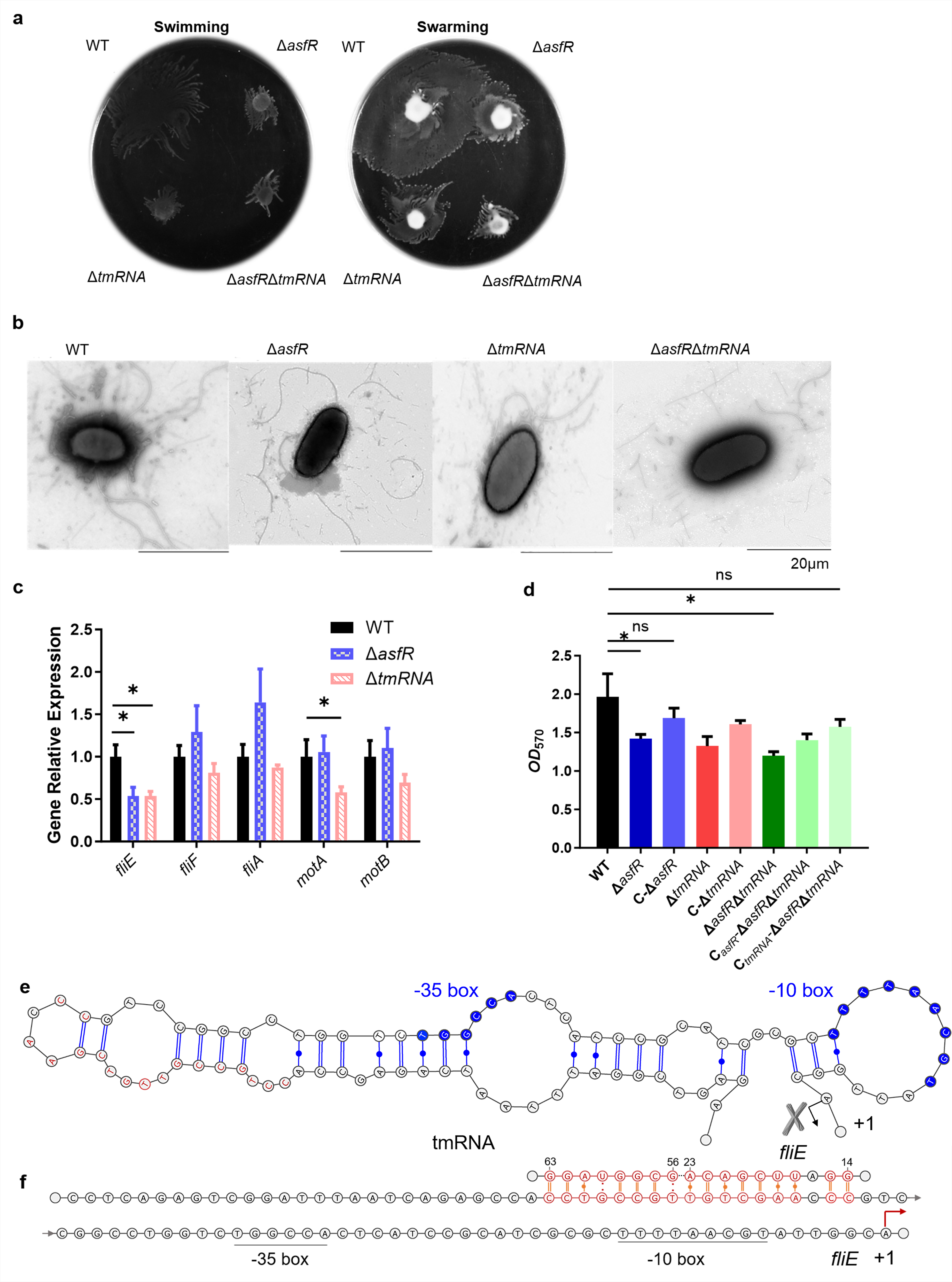

Supplement: Figures.zip [file KVIR_A_2602247_SM0847.zip › Figures/Figure3.tif]

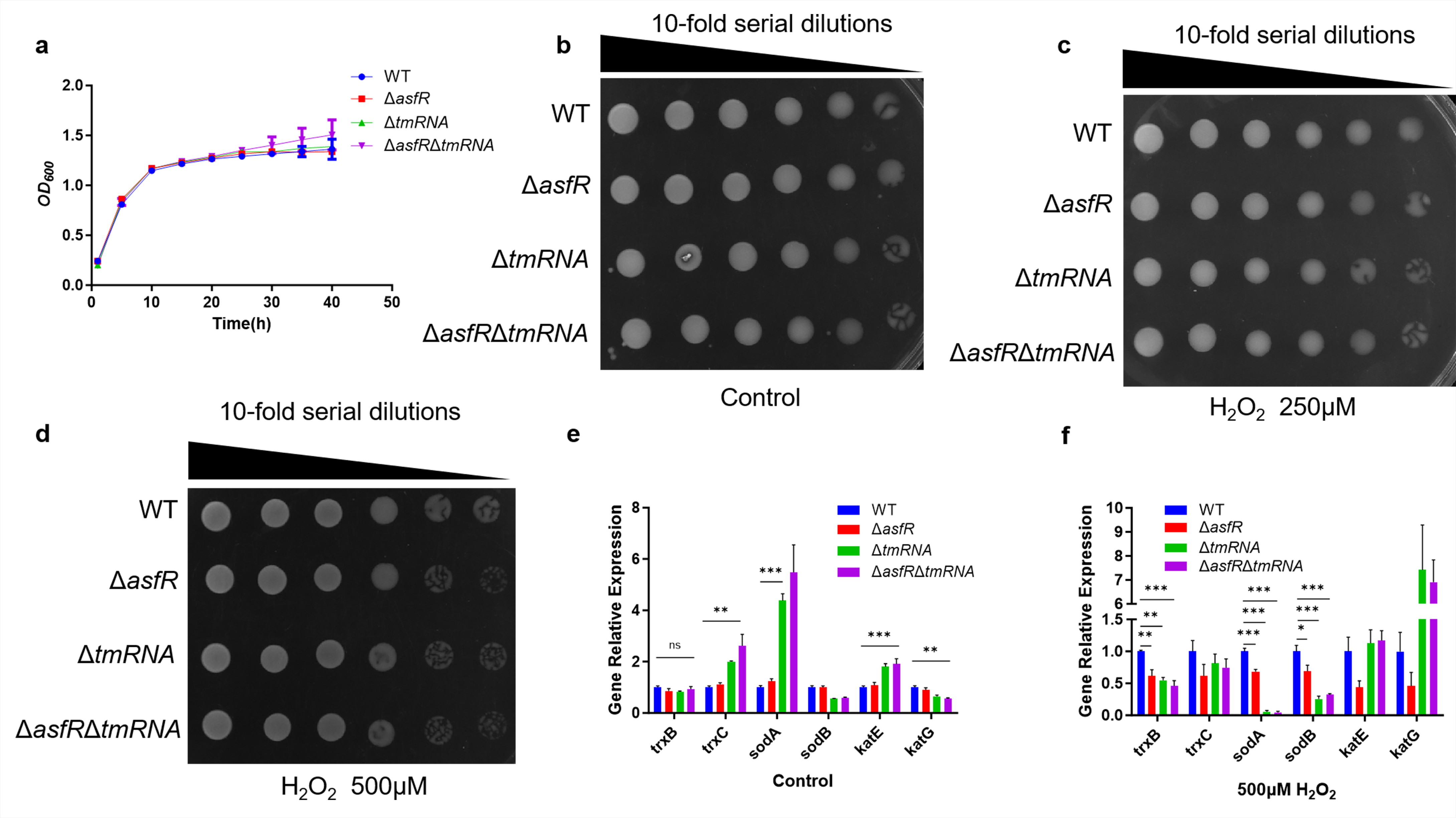

Supplement: Figures.zip [file KVIR_A_2602247_SM0847.zip › Figures/Figure4.tif]

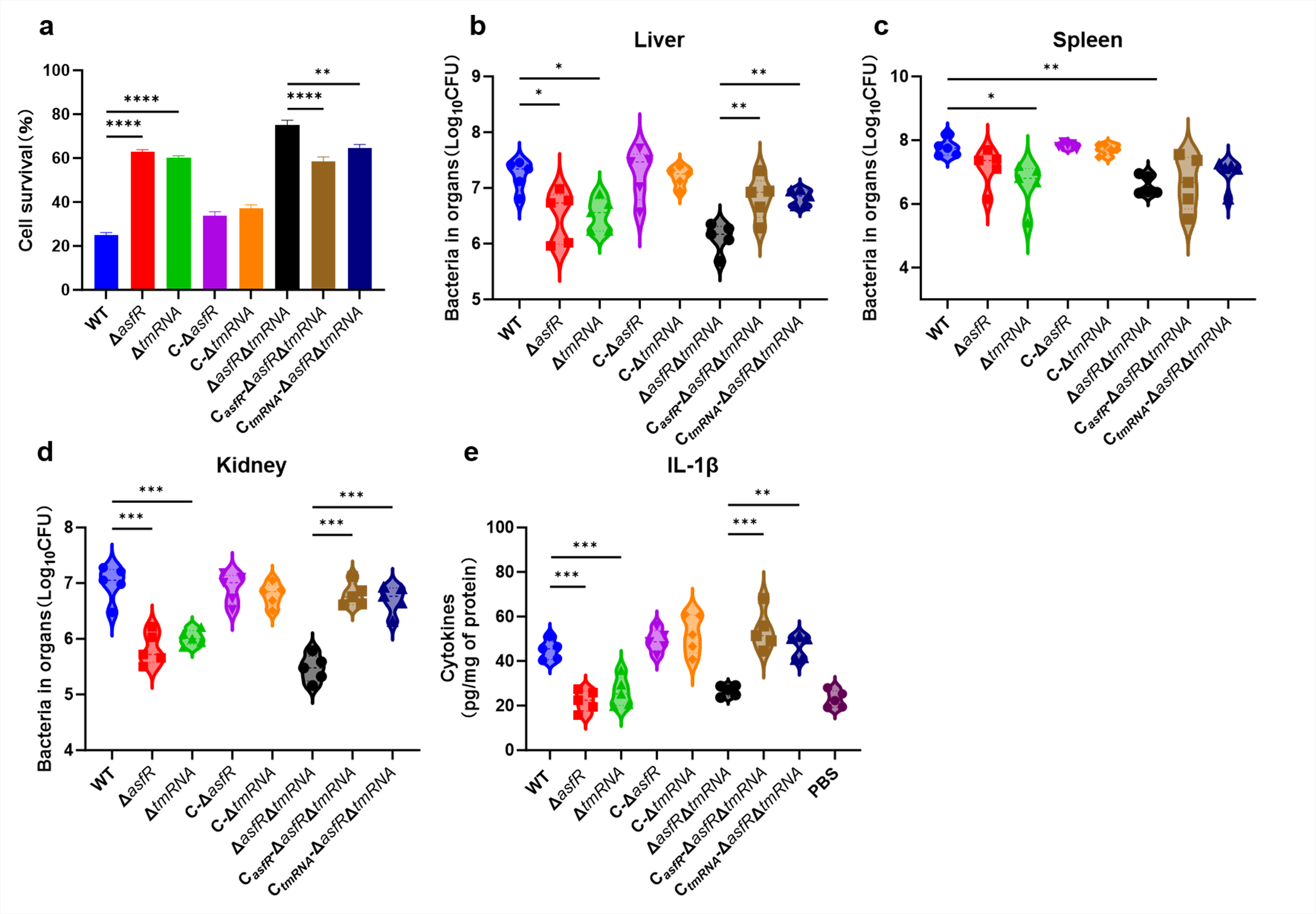

Supplement: Figures.zip [file KVIR_A_2602247_SM0847.zip › Figures/Figure5.tif]

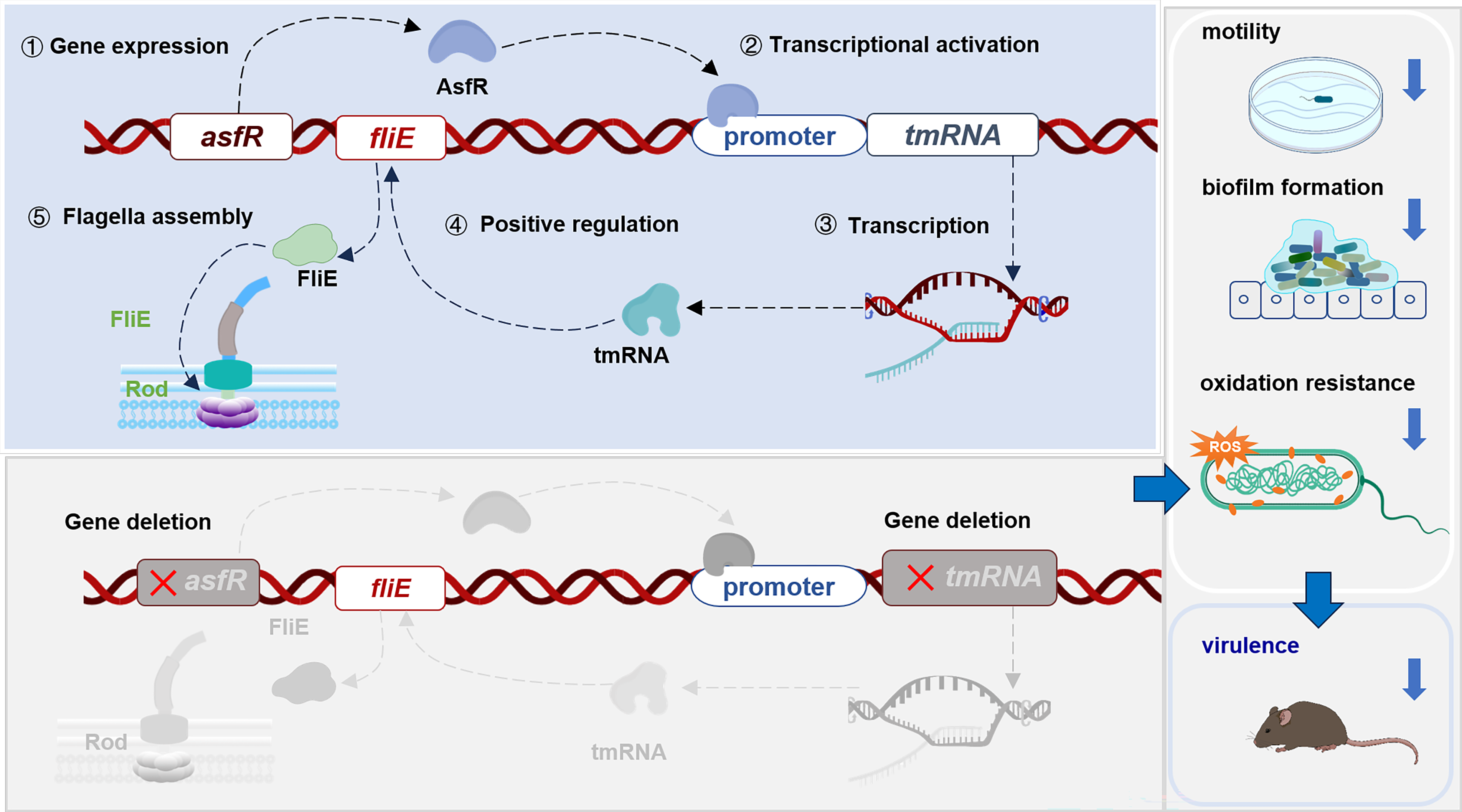

Supplement: Figures.zip [file KVIR_A_2602247_SM0847.zip › Figures/Figure6.tif]

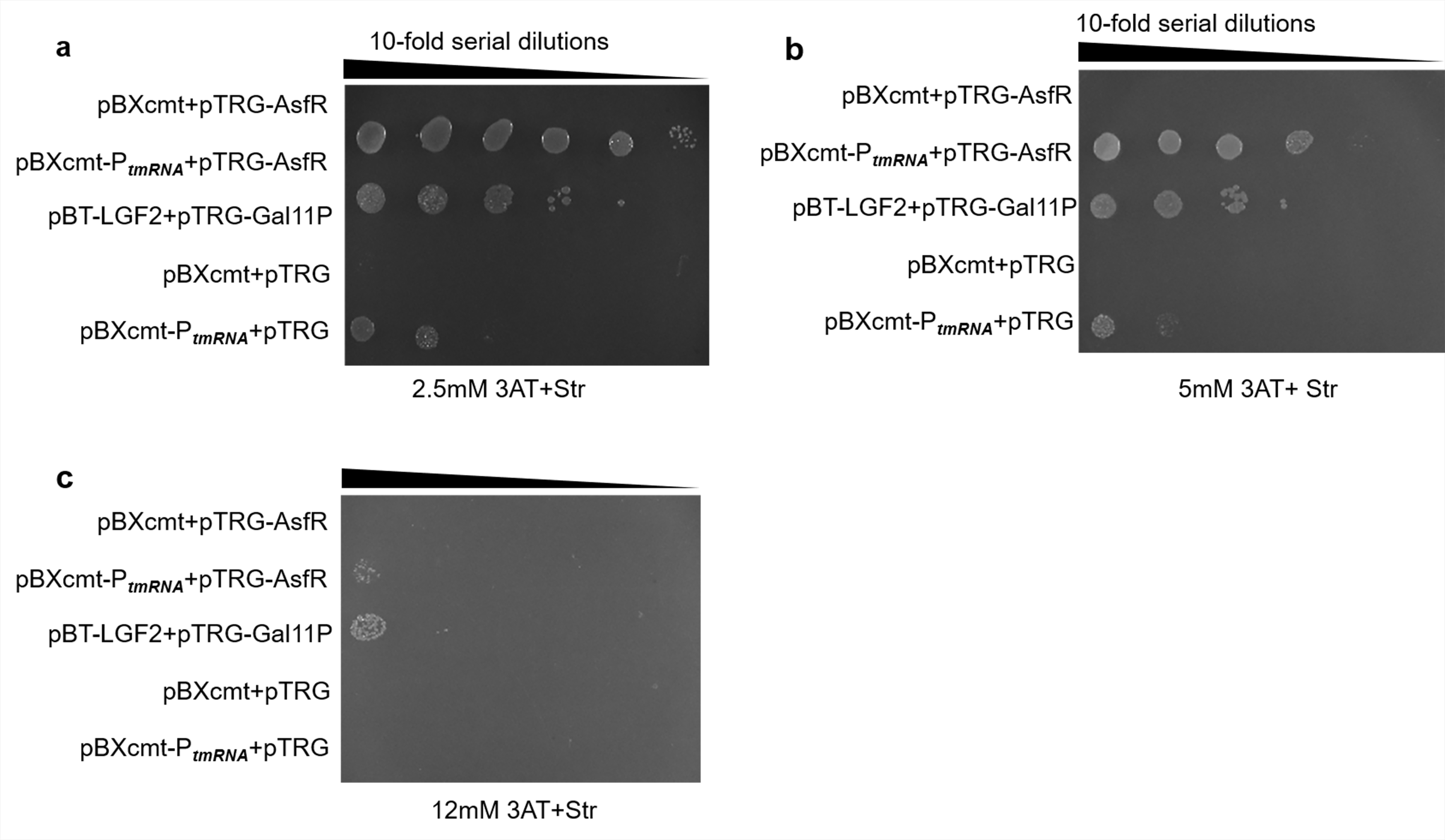

Supplement: Figures.zip [file KVIR_A_2602247_SM0847.zip › Figures/FigureS1.tif]

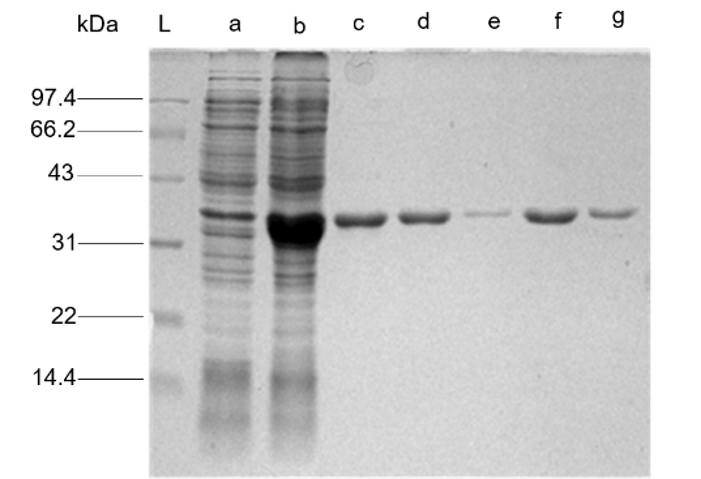

Supplement: Figures.zip [file KVIR_A_2602247_SM0847.zip › Figures/FigureS2.tif]

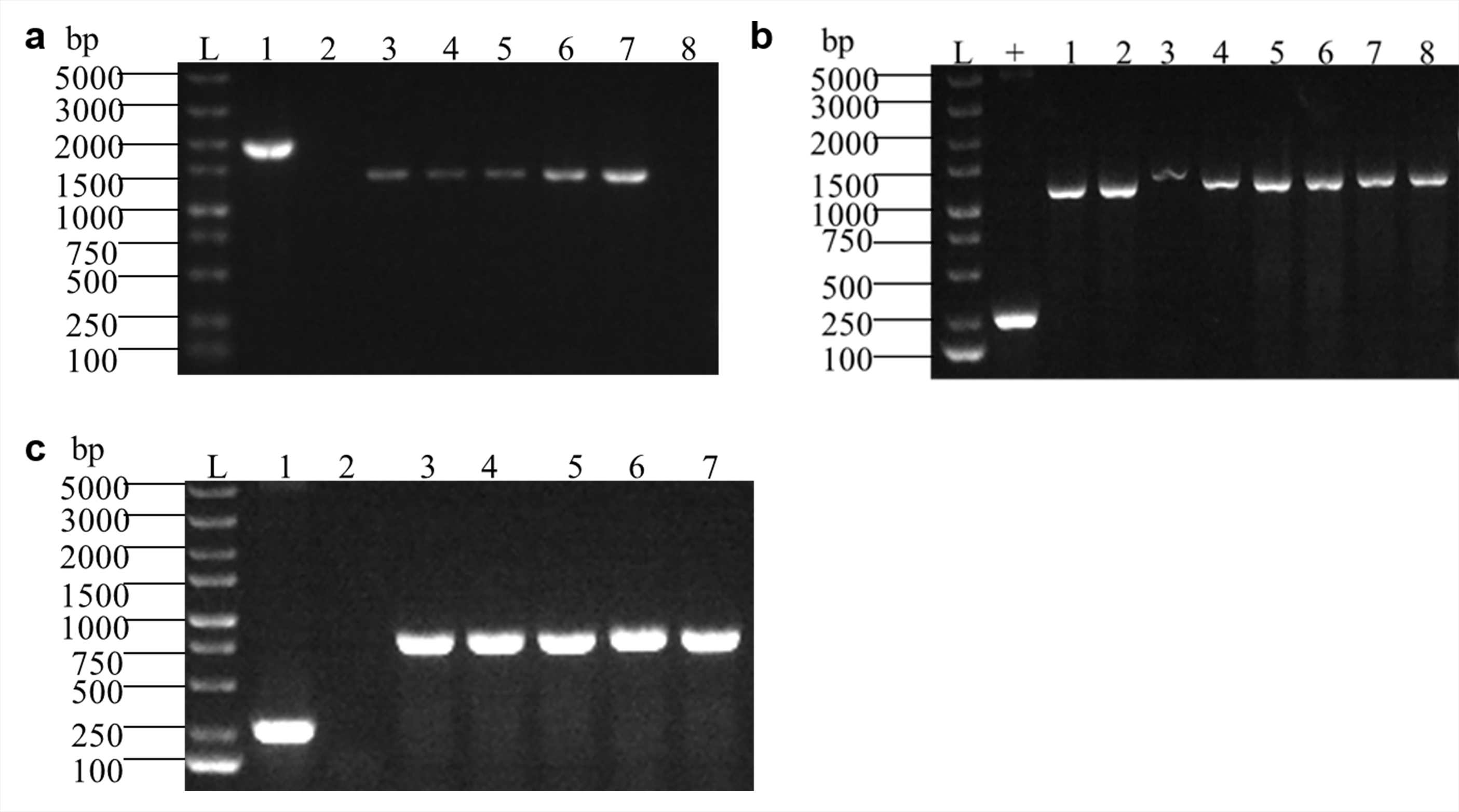

Supplement: Figures.zip [file KVIR_A_2602247_SM0847.zip › Figures/FigureS3.tif]

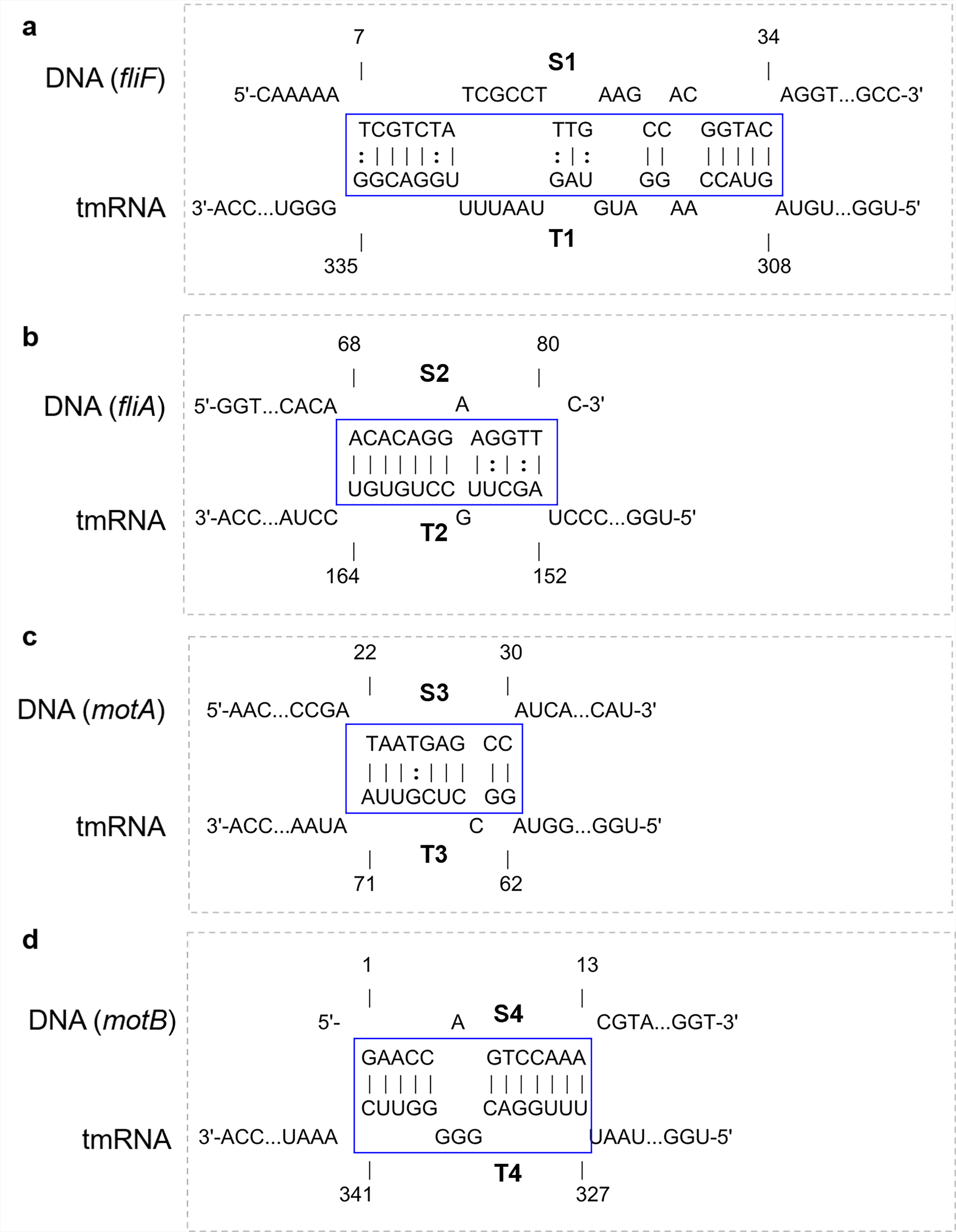

Supplement: Figures.zip [file KVIR_A_2602247_SM0847.zip › Figures/FigureS4.tif]

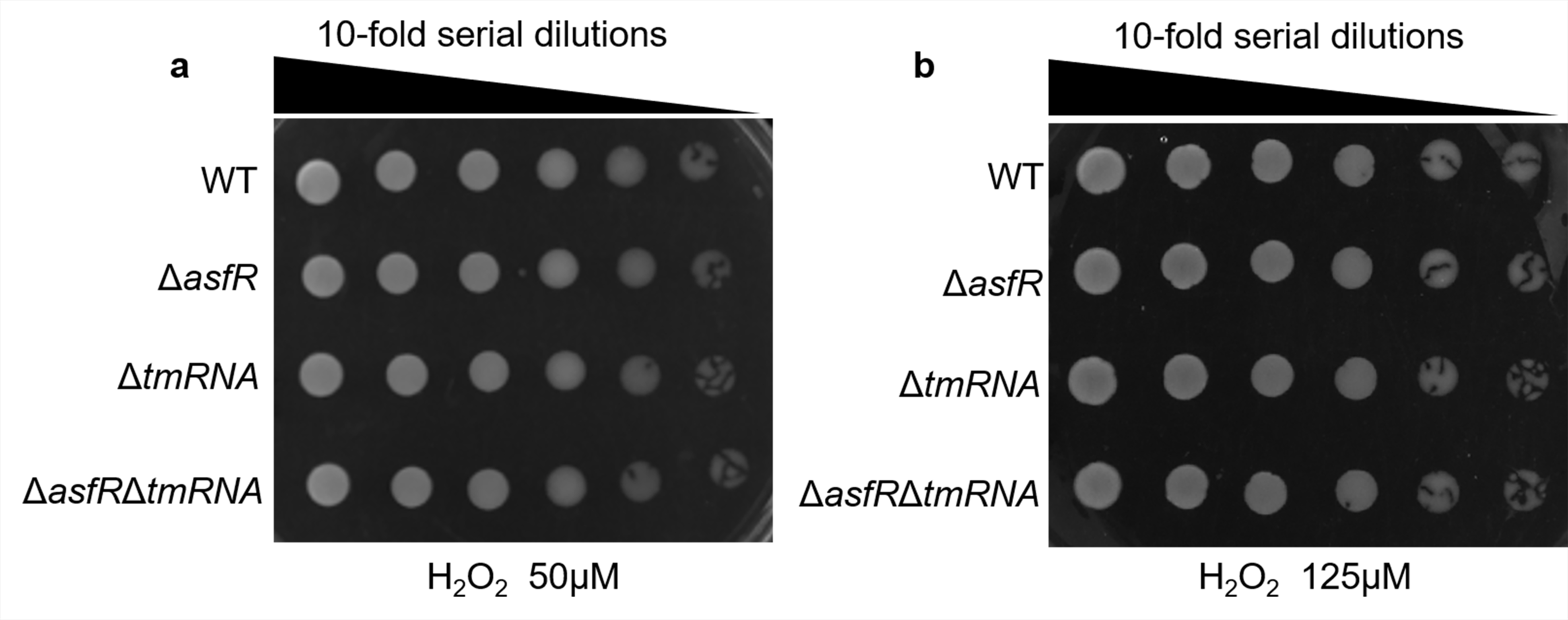

Supplement: Figures.zip [file KVIR_A_2602247_SM0847.zip › Figures/FigureS5.tif]

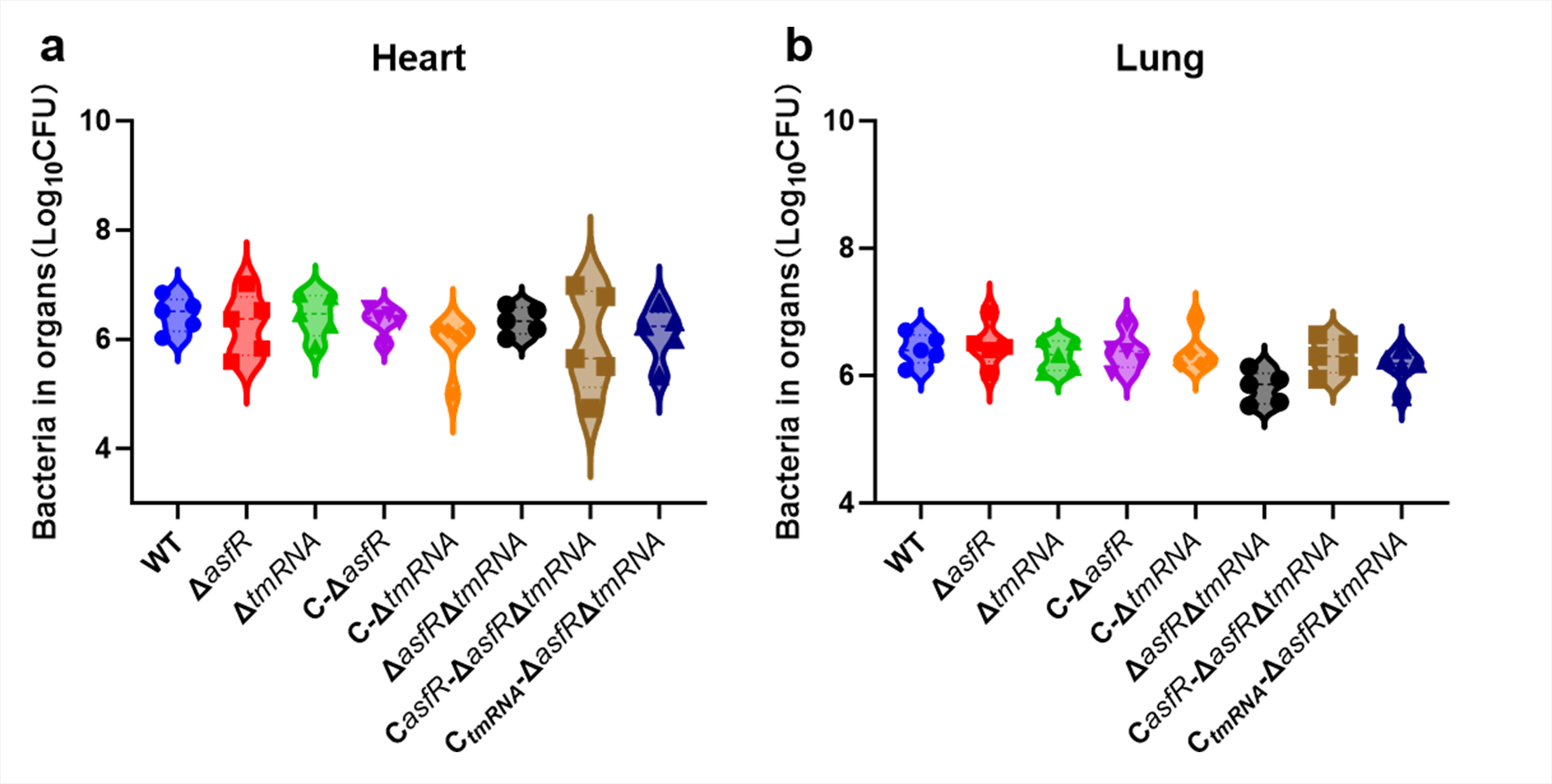

Supplement: Figures.zip [file KVIR_A_2602247_SM0847.zip › Figures/FigureS6.tif]
